# Unlocking the potential of atractylenolide II: Mitigating non-alcoholic fatty liver disease through farnesoid X receptor-endoplasmic reticulum stress interplay

**DOI:** 10.1016/j.jpha.2025.101318

**Published:** 2025-04-21

**Authors:** Ming Gu, Zhiwei Chen, Yujun Chen, Yiping Li, Hongqing Wang, Ya-ru Feng, Peiyong Zheng, Cheng Huang

**Affiliations:** a Institute of Digestive Disease, Longhua Hospital, Shanghai University of Traditional Chinese Medicine, Shanghai 200032, China; b School of Pharmacy, Shanghai University of Traditional Chinese Medicine, Shanghai 201203, China

**Keywords:** Endoplasmic reticulum stress, Farnesoid X receptor, Sarco/endoplasmic reticulum Ca^2+^ ATPase 2, Non-alcoholic fatty liver disease, atractylenolide II

## Abstract

Evidences indicate that farnesoid X receptor (FXR) activation mitigates non-alcoholic fatty liver disease (NAFLD) by reducing endoplasmic reticulum (ER) stress. However, the mechanisms underlying FXR-ER stress interactions in combating NAFLD remain obscure. Moreover, few phytochemicals have been noted to improve NAFLD through this pathway. Here, we found that FXR activation directly induces the transcription of sarco/endoplasmic reticulum Ca^2+^ ATPase 2 (SERCA2), which acts as an ER stress repressor. This process leads to the dephosphorylation of the eukaryotic translation initiation factor 2 subunit α (eIF2α) within hepatocytes, consequently alleviating ER stress. Furthermore, through drug binding assays, luciferase reporter gene testing, gene expression analysis and biochemical evaluation, we identified the phytochemical atractylenolide II (AT-II) as a novel FXR agonist that effectively triggers SERCA2 activation. Our results showed AT-II effectively supresses accumulation of lipids and ER stress in palmitic acid-induced hepatocytes. In *in vivo* experiments, we demonstrated that AT-II attenuates fatty liver in diet- or chemical-induced NAFLD mouse models. Additionally, we showed that AT-II corrects diet-induced obesity, serum dyslipidemia, metabolic complications, and insulin resistance. Mechanistically, AT-II reduces ER stress, lipogenesis and inflammation and improves hepatic insulin signaling through stimulation of the hepatic FXR-SERCA2-eIF2α axis in mice. This conclusion was further reinforced by *Serca2* knockdown both *in vivo* and *in vitro*, as well as FXR silencing in hepatocytes. Our findings provide new insights into the FXR-ER stress interplay in the control of NAFLD and suggest the potential of AT-II as an FXR agonist for the treatment of NAFLD through SERCA2 activation.

## Highlights

• FXR activation inhibits hepatic ER stress by inducing SERCA2 transcriptionally.
• Atractylenolide II (AT-II) is identified as a novel FXR activator.
• AT-II potentiates SERCA2 in hepatocytes by activating FXR.
• AT-II attenuates ER stress and NAFLD via promoting FXR-SERCA2 axis.
• Dephosphorylation of eIF2α by SERCA2 is involved in AT-II's anti-NAFLD.

## Graphical abstract

**Figure:**
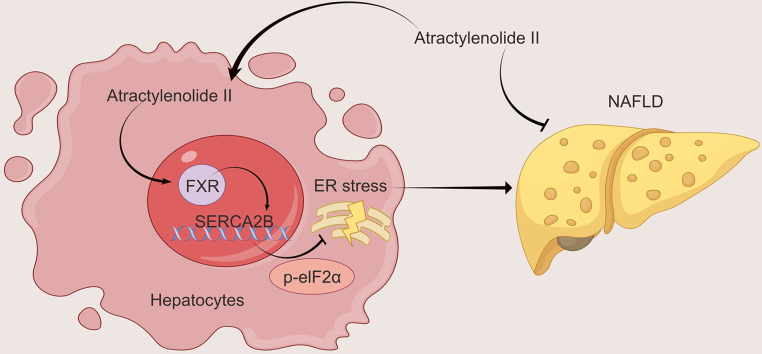

## 1 Introduction

Non-alcoholic fatty liver disease (NAFLD), now referred to as metabolic dysfunction-associated steatotic liver disease (MASLD), is a widespread chronic liver condition that affects more than one-third of the global population [1]. NAFLD can progress to non-alcoholic steatohepatitis (NASH) and liver fibrosis, which may lead to liver cancer and cirrhosis [2]. There is a significant association between NAFLD and conditions such as type 2 diabetes mellitus (T2DM), obesity, and hyperlipidemia [3]. However, given the ambiguity of pathogenesis and the absence of effective pharmacological treatments, the prevalence of NAFLD continues to increase worldwide [1]. Consequently, understanding the pathogenesis of NAFLD and developing effective drugs are essential to improve disease management.

Chronic endoplasmic reticulum (ER) stress triggers the unfolded protein response (UPR), which plays a vital role in obesity, diabetes and NAFLD [4]. Individuals with metabolic disorders frequently exhibit ER stress in their energy metabolism tissues, exacerbating these conditions [5]. ER stress contributes to the progression of NAFLD by promoting dysglycolipidemia, leptin resistance, insulin resistance (IR), apoptosis, oxidative stress, and inflammation [6]. On the other hand, interventions that target ER stress pathways, both genetic and pharmacological, have shown potential in mitigating NAFLD [7].

The sarco/endoplasmic reticulum Ca^2+^ ATPase 2 (SERCA2) pump, a type of P-type ATPase, is essential for maintaining the equilibrium of ER Ca^2+^ reserves [8]. It plays a crucial role in regulating ER stress and is linked to metabolic disorders [[9], [10], [11]]. In obese mice, diminished SERCA2 expression in the liver correlates with hepatic ER stress, but restoring the levels and activity of SERCA2 can mitigate this stress and improve glucose tolerance [[12], [13], [14]]. Additionally, enhancing SERCA2 functionality can improve pancreatic β-cell performance [9,15], support the development of beige adipose tissue, and increase energy expenditure [16], highlighting its potential as a therapeutic target for treating NAFLD.

Nuclear receptor farnesoid X receptor (FXR) is a key member of the bile acid receptor family involved in regulation of various physiological processes by transcriptionally controlling gene expression [17]. Beyond its involvement in bile acid metabolism, activating FXR is critical for managing energy metabolism disorders and NAFLD [18,19]. FXR influences glucose production and transport, enhances insulin sensitivity, reduces lipid synthesis, stimulates lipolysis, and possesses anti-inflammatory and anti-fibrotic effects [18]. Additionally, there is evidence suggesting that FXR activation may help mitigate ER stress [[20], [21], [22]]. However, the precise mechanisms by which liver FXR interacts with ER stress signaling remain unclear. Therefore, further exploration of FXR's biological roles and the development of new agonists are promising avenues for advancing treatment and prevention strategies for NAFLD.

Atractylenolide II (AT-II), a diterpene lactone mainly derived from *Atractylodes macrocephala Koidzumi* (AMK), exhibits a range of medicinal properties, including reducing inflammation, acting as an antioxidant, and combating tumors, bacteria, and modulating immune functions [23,24]. Recent research has investigated the potential of AT-II in improving anomalies in glucose-lipid metabolism, addressing obesity, and decreasing liver steatosis [24,25]. However, its pharmacological effects and the molecular mechanisms underlying its role in ER stress-related fatty liver disease remain incompletely understood.

The current study demonstrated that activation of FXR can increase SERCA2 gene expression, thereby reducing ER stress in hepatocytes. Furthermore, we found that AT-II acts as a natural activator of the FXR-SERCA2 pathway, effectively mitigating intrahepatic ER stress in both cell culture and animal models. Animal studies additionally revealed that AT-II ameliorates conditions associated with ER stress-related fatty liver disease, such as obesity and disrupted glucose-lipid metabolism. We also observed that hepatic SERCA2 is vital to AT-II's positive impact on liver steatosis, as it reverses ER stress and restores insulin signaling. This study provides new insights into the interactions between FXR activation, ER stress, and NAFLD, presenting promising avenues for therapeutic intervention.

## 2 Methods

### 2.1 Materials

For the *in vitro* experiments, AT-II (Cat# B20055, Yuanye Biol Comp, Shanghai, China) was dissolved in dimethyl sulfoxide (DMSO; Cat# D8418, Sigma-Aldrich, St Louis, MO, USA) to achieve a concentration of 50 mM. Other reagents, including GW4064 (Cat# G5172), tunicamycin (TM; Cat# 654380), insulin (Cat# 10908), dexamethasone (Cat# 265005), insulin-transferrin-selenium (ITS; Cat# I3146), chenodeoxycholic acid (CDCA; Cat# C9377), obeticholic acid (OCA; Cat# SML3096), and palmitic acid (PA; Cat# P0500), were sourced from Sigma-Aldrich. In the *in vivo* studies, mouse models of obesity and NASH were established using high-fat diets (HFD 60%; Cat# D12492, Research Diets, New Brunswick, NJ, USA) and methionine and choline-deficient l-amino acid diets (MCD 20%; Cat# A02082002B, Research Diets), respectively. Normal control mice were fed with either chow diets (Cat# D12450B) or methionine and choline-sufficient l-amino acid diets (MCS; Cat# A02082003B) diets, both of which were procured from Research Diet.

### 2.2 Culture and treatment of cells

The human HepG2 and AML-12 mouse liver cell lines were supplied by the Shanghai Institute of Cell Biology (Shanghai, China). HepG2 cells were cultured in high-glucose dulbecco's modified eagle medium (DMEM; Cat# 11965, Thermo Fisher Scientific, Shanghai, China) supplemented with 10% fetal bovine serum (FBS; Cat# A5670701, Thermo Fisher Scientific) and 100 units/mL of streptomycin-penicillin (Cat# C0222, Beyotime, Shanghai, China). AML-12 cells were grown in DMEM-F12 (Cat# 113200, Thermo Fisher Scientific) enriched with 10% FBS, 0.1 mM dexamethasone, and a cocktail of ITS. Both cell lines were maintained at 37 °C under a 5% CO_2_ atmosphere. To establish *in vitro* models of ER stress and to assess AT-II treatment, HepG2 or AML-12 cells were treated with either bovine serum albumin (BSA; Cat# 36104 ES, Yeasen, Shanghai, China), BSA-conjugated PA (400 μM), or TM (2 μg/mL), followed by exposure to AT-II for 24 h.

### 2.3 Cell counting kit 8 (CCK8) assays

The CCK8 assay was conducted to evaluate cytotoxic activity of HepG2 cells following treatment of AT-II using a CCK8 kit (Cat# C0037, Beyotime) following the manufacturer's instructions. In preparation for CCK8 detection, HepG2 cells were seeded into a 96-well plate at a density of 4 × 10^5^ cells/mL. After 24-h cell culture, cells were incubated with AT-II at indicated concentration for another 24 h. Then, 10 μL of CCK8 reagent (5 mg/mL) was incorporated into the each well 1 h ahead of the analysis. The absorbance values at 450 nm was read with a microplate reader (Synergy™ 4, BioTek, Winooski, VT, USA). Three independent repetitions of the experiments were performed.

### 2.4 Quantitative real-time polymerase chain reaction (qRT-PCR)

Total RNA was extracted from both tissues and cultured cells using the TRIzol reagent and subsequently reverse transcribed into cDNA using a cDNA Synthesis Kit (Cat# RK20400, Abclonal, Wuhan, China). The synthesized cDNA was analyzed by qRT-PCR, employing the Maxima SYBR Green qPCR Master Mix (Cat# RK20404, Abclonal). Gene expression levels were normalized to actin beta (*ACTB*). The data were shown as relative messenger RNA (mRNA) levels with three independent repetitions. Relative mRNA levels were normalized to the mean of the corresponding negative control groups or model vehicle groups. The primers sequences have been illustrated in Tables S1 and S2.

### 2.5 Western blotting analysis

Total protein was extracted using a protein lysis kit (Cat# 20115 ES, Yeasen). Nuclear and cytoplasmic proteins from liver tissues were isolated using a NE-PER™ Kit (Cat# 78833, Thermo Fisher Scientific). The protein samples were immunoblotted as previously described [22]. The expression of specific proteins was detected using antibodies against ACTB (Cat# 3700, Cell Signaling Technology, Danvers, MA, USA), FXR (Cat# sc-25309, Santa Cruz Biotechnology, Santa Cruz, CA, USA), SERCA2 (Cat# A1097, ABclonal), small heterodimer partner (SHP; Cat# A1836, ABclonal), protein kinase, endoplasmic reticulum kinase (PERK; Cat# 20582-1-AP, Proteintech Group, Wuhan, China), phosphorylated PERK (p-PERK; Cat# 3179, Cell Signaling Technology), eukaryotic translation initiation factor 2 subunit α (eIF2α; Cat# 9722, Cell Signaling Technology), phosphorylated eIF2α (p-eIF2α; Cat# 3398, Cell Signaling Technology), glucose-regulated protein 78 (BIP; Cat# 3177, Cell Signaling Technology), activating transcription factor 4 (ATF4; Cat# 10835-1-AP, Proteintech Group), C/EBP homologous protein (CHOP; Cat# 15204-1-AP, Proteintech Group), AKT serine/threonine kinase (AKT; Cat# 9272, Cell Signaling Technology), phosphorylated AKT (p-AKT; Cat# 9271, Cell Signaling Technology), c-Jun *N*-terminal kinase (JNK; Cat# 66210-1-ig, Proteintech Group), phosphorylated JNK (p-JNK; Cat# 4668, Cell Signaling Technology) and Histone H3 (H3; Cat# 4499, Cell Signaling Technology). Protein blots were visualized using Chemi-Lumin One Ultra (Tanon Science & Technology Co., Ltd., Shanghai, China) with a chemiluminescence detection kit (Cat# 34577, Thermo Fisher Scientific). All protein quantification data were expressed as relative band intensities and normalized to the mean of the negative control or model vector groups, repeated independently three times.

### 2.6 Cellular thermal shift assay (CETSA)

HepG2 cells were lysed using liquid nitrogen by 5 cycles of freeze-thawing. The supernatant of the cell lysate was then collected via centrifugation. This supernatant was incubated for 30 min at room temperature with either 0.1% DMSO or AT-II (100 mM). Subsequently, samples from both groups were transferred to polymerase chain reaction (PCR) tubes and subjected to a temperature gradient (50, 52, 54, 56, 58, 60, 62, 64, 66, and 68 °C) for 3 min, followed by 3 min at room temperature. Afterward, samples were spun down in a low-temperature centrifugation, and the resulting supernatants were collected for analysis via sodium dodecyl sulfate polyacrylamide gel electrophoresis (SDS-PAGE).

### 2.7 Time-resolved fluorescence resonance energy transfer (TR-FRET) analysis

A LanthaScreen™ TR-FRET FXR coactivator assay was carried out as per the manufacturer's guidelines (Cat# A15140, Invitrogen, Carlsbad, CA, USA) to assess AT-II's binding to FXR-ligand binding domain (FXR-LBD), using GW4064 as a positive control.

### 2.8 Luciferase reporter assays

To determinate FXR transcription activity, HEK293T cells (Shanghai Institute of Cell Biology, Shanghai, China) were plated in 48-well plates and cultured for 24 h prior to transfection. Then, 2 μg of each expression plasmids for phFXR, phRXR, FXR-dependent reporter (EcRE-Luc), and 0.2 μg of internal interference plasmid pREP7 Renilla luciferase were co-transfected into HEK293T cells by using the lipofectamine 3000 kit (Cat# L3000, Invitrogen). In detail, a transfection reaction was prepared by mixing 6 μg of total plasmids in serum-free DMEM with 12 μL of P3000 Reagent and 10 μL of Lipofectamine 3000 Reagent from the kit. The DNA-lipid complexes were added to cells in each well for 24 h, and then replaced with fresh DMEM or treatment with 0.1% DMSO, GW4064 (5 μM) and various concentrations of AT-II for an additional 24 h.

For the SERCA2 promoter luciferase (SERCA2-Luc) reporter assay, potential FXR binding sites on the SERCA2 promoter (Site#1, #2, and #3, detailed in Table S3) were identified using JASPAR 3.0. The SERCA2 promoter sequence (ranging from −3000 to +167, which includes Site#1, #2, and #3) was amplified from HepG2 cell genomic DNA. Mutations combining Site#2 and #3, Site#1 and #3, and Site#1 and #2 were introduced into the SERCA2 promoter and cloned into pGL3-Basic vector (Cat# E1751, Promega, Madison, WI, USA), sourced from Sangon Biotech (Shanghai, China). The 2 μg of wild-type or 2 μg of mutant SERCA2-Luc plasmid was co-transfected with 0.2 μg of pREP7 into 48-well plate cultures of HepG2 cells with or without 2 μg of phFXR overexpression for 24 h using the Lipofectamine 3000 kit according to the manufacturer's instructions. Treatments with 0.1% DMSO, CDCA (10 μM), OCA (10 μM), and various concentrations of GW4064 or AT-II were administered to the cells for an additional 24 h.

After a 48-h transfection, the cells were lysed using a passive lysis buffer (Cat# E1941, Promega). Luciferase activity, indicated by relative light unit (RLU), was measured using the Dual-Luciferase Reporter gene assay kit (Cat# 11405 ES, Yeasen). This was achieved by incorporating luciferase detection reagents into the cell lysate. Renilla luciferase activity served as a normalization control for transfection efficiencies. All data of RLU were normalized to the mean of the control group. All transfection assays were carried out in triplicate with three independent repeats.

### 2.9 Chromatin immunoprecipitation (ChIP) assay

Briefly, HepG2 cells were transfected with a human FXR overexpression plasmid or a control plasmid for 48 h. At 37 °C, proteins bound to DNA from 1 × 10^7^ cells were crosslinked for 30 min with 1% formaldehyde. Subsequently, the cells were lysed and the DNA was sheared into approximately 500 bp fragments using an ultrasonic processor. The samples underwent pre-clearance with protein G agarose beads before being immunoprecipitated overnight at 4 °C using either an anti-FXR antibody (Cat# sc-25309, Santa Cruz Biotechnology) or a control immunoglobulin G (IgG) antibody (Cat# 2729, Cell signaling Technology). DNA from the extracted chromatin-protein/DNA complexes was then purified using spin columns. Following this, qRT-PCR was conducted using specific primers (detailed in Table S4) targeting the SERCA2 gene promoter region.

### 2.10 Molecular docking

The crystal structure of the FXR protein, obtained from the Research Collaboratory for Structural Bioinformatics (RCSB) Protein Data Bank (PDB) under PDB code 1OT7, was used as the target for subsequent molecular docking studies carried out with Molecular Operating Environment (MOE) software. The MOE's QuickPrep module prepared the protein by adding hydrogens and minimizing energy. The Wash module of MOE prepared AT-II, retrieved from PubChem (https://pubchem.ncbi.nlm.nih.gov), by protonating it and generating its 3D structure. A conformational search was conducted to explore potential conformations. The FXR protein, particularly focusing on the 3-deoxy-CDCA binding site, served as the receptor, and a selected AT-II conformation was used as the ligand for molecular docking.

### 2.11 Intracellular lipids staining

HepG2 cells were co-incubated with 0.5 mM PA and AT-II for 24 h. Following this, cells were stained using boron-dipyrromethene (BODIPY) 493/503 according to the manufacturer's protocol (Cat# D3922, Invitrogen) to visualize intracellular lipid droplets. Subsequently, the nuclei were counterstained with 5 μg/mL of 4′,6-diamidino-2-phenylindole (DAPI; Cat# 40728 ES, Yeasen). Imaging was then performed to assess the lipid droplet accumulation.

### 2.12 Detecting Ca^2+^ ATPase activity

The cell samples were used for Ca^2+^ ATPase activity assay after repeated freeze-thawing. The intracellular Ca^2+^ ATPase activity was measured using an Ultramicro Ca^2+^ATPase Diagnostic Kit (Cat# A070-4-2, Nanjing Jiancheng, Nanjing, China). This assay quantifies the inorganic phosphorus released during the conversion of adenosine triphosphate (ATP) to adenosine diphosphate (ADP), utilizing molybdenum blue spectrophotometry at a wavelength of 660 nm. The results data were expressed as relative Ca^2+^ ATPase activity. Data normalization was performed using the mean of the DMSO control groups. The experimental results represent three independent technical replications.

### 2.13 RNA interference (RNAi) experiments

Human *FXR*-siRNA, mouse *Serca2*-siRNA, and a negative control siRNA were synthesized (Genomeditech, Shanghai, China). HepG2 and AML-12 cells were grown in six-well plates at a density of 2 × 10^5^ cells/well using antibiotic-free culture medium, and cultured at 37 °C. After a 24-h incubation, human *FXR*-siRNA, mouse *Serca2*-siRNA, and a negative control siRNA were proportionally mixed with Lipofectamine 2000 Reagent (Cat#11668, Invitrogen) at 1:1 ratio using serum-free DMEM dilution, and the mixtures were incubated at room temperature for 5 min before transfection. These siRNAs (50 nM) were then transfected into HepG2 and AML-12 cells grown to 70% confluence through addition of these transfection complexes to each well of cells. After a 48-h transfection, the efficiency of the knockdown was verified through Western blot analysis. The RNAi experiments were completed by three independent replicates and ACTB was used as an internal control. Relevant gene expression data were normalized to the mean of the negative si-control groups. The siRNA sequence were shown in Table S5.

### 2.14 Animals and treatments procedures

The animal study protocol was approved by the Shanghai University of Traditional Chinese Medicine (Approval number: PZSHUTCM211115002) and conducted in accordance with the ARRIVE guidelines [26]. Male C57BL/6 mice, aged 8 weeks, were provided by the SLAC Laboratory (Shanghai, China). Following an acclimatization period, the mice were housed in a specific pathogen-free (SPF) environment with a 12-h light/12-h dark cycle, at temperatures of 22–23 °C and 60% ± 10% relative humidity. To induce diet-induced obesity (DIO), mice were fed either a standard chow diet (*n* = 7) or a HFD for 12 weeks. Obese mice were then randomly divided into groups: one receiving HFD alone (*n* = 7) and the others receiving HFD supplemented with varying concentrations of AT-II powder (25, 50, or 100 mg/100 g diet, *n* = 7 for each concentration). The high dose of AT-II (100 mg/100 g diet) corresponded to 60 mg/kg in mice and was selected based on a prior study that showed no acute or chronic *in vivo* toxicity [25]. Food intake was monitored and calculated every two days based on the amount of food consumed over a 24-h period. The treatment period lasted for 6 weeks.

To induce NASH in male C57BL/6J mice via MCD, mice (weighing approximately 20 ± 2 g) were initially fed either a MCS diet as a normal control or an MCD diet to induce NASH. After 4 weeks on the MCD diet, mice were randomly assigned to either continue on the MCD diet or to receive an MCD diet supplemented with AT-II powder. This feeding regimen continued for an additional 4 weeks prior to the mice being sacrificed. Each experimental group consisted of 7 mice.

To construct a TM-induced ER stress mouse model, male C57BL/6 mice, aged 10 weeks, were randomly divided into three groups: control, TM, and TM + AT-II. For 7 consecutive days, the TM and TM + AT-II groups received intraperitoneal injections of TM (1 mg/kg), while the TM + AT-II group also received AT-II (25 mg/kg/day). The control group received equivalent volumes of saline. Thirty hours after the final injection, mice were anesthetized and euthanized following fasting blood glucose assessments.

Adeno-associated virus 8 (AAV8) vectors carrying a mouse *Serca2* short hairpin RNA (shRNA) (AAV8-sh*Serca2*) or a negative control shRNA (AAV8-shNC) were procured from Genomeditech. The shRNA sequences are shown in Table S6. Male C57BL/6J mice, 8 weeks old, received tail vein injections of either AAV8-shNC or AAV8-sh*Serca2* (1 × 10^11^ viral genomes per mouse) after being fed a HFD for 12 weeks. Subsequently, the mice continued on the HFD with or without AT-II supplementation (100 mg/100 g diet) for an additional 6 weeks. Each experimental group included 6 mice.

After each animal study, the mice were humanely euthanized using 20% urethane (Cat# U2500, Sigma-Aldrich) anesthesia. Cardiac blood samples were then collected, and various tissues were extracted for subsequent detailed analysis as outlined in the study protocols.

### 2.15 Metabolic expenditure measurements

To evaluate energy metabolism, mice from the DIO experiment were acclimated in CLAMS metabolic PhenoCages (Columbus Instruments, Columbus, OH, USA) for 24 h. They were then monitored continuously over a 36-h period, during a regular light-dark cycle (light from 6:00 to 18:00 and dark from 18:00 to 6:00). Measurements taken included oxygen consumption (VO_2_), carbon dioxide production (VCO_2_), heat production, and locomotor activity. The data collected from the last 24 h of this period were analyzed.

### 2.16 Biochemical examination of blood serum and hepatic tissue

Mouse serum levels of total cholesterol (TC), low-density lipoprotein cholesterol (LDL-c), high-density lipoprotein cholesterol (HDL-c), triglycerides (TG), lactic dehydrogenase (LDH), alanine aminotransferase (ALT), and aspartate transaminase (AST) were assessed using an automatic analyzer (Hitachi 7020, Hitachi, Tokyo, Japan). Additionally, liver tissue samples weighing 50 mg each were homogenized using chloroform-methanol based on the Folch method [27] for hepatic TG and TC extraction, or saline for extracting hepatic total bile acids (TBA) and malondialdehyde (MDA). The levels of TG, TC, TBA, and MDA in the liver tissues were then determined using commercially available kits (Nanjing Jiancheng), following the instructions provided with each kit.

### 2.17 Tolerance tests

After 12-h fasting, mice were subjected to a fasting blood glucose measurement. They then received an intraperitoneal injection of glucose (1 g/kg body weight) to initiate glucose tolerance tests (GTTs), with blood glucose levels measured at 15, 30, 60, and 90-min intervals thereafter. For insulin tolerance tests (ITTs), mice were administered 0.75 U/kg of insulin intraperitoneally. Glucose levels were subsequently measured by puncturing the tail vein at intervals of 0, 15, 30, 60, 90, and 120 min post-injection.

### 2.18 Histological evaluation

Liver and adipose tissues were fixed in 4% formalin before being embedded in paraffin. The sections of 5 μm were prepared and stained with hematoxylin and eosin (H&E), Sirius Red, and Periodic acid-Schiff (PAS). The NAFLD activity score (NAS) of the liver sections were conducted by a hepatopathologist according to previous description [28]. Additionally, frozen liver sections underwent Oil Red O (ORO) staining using standard protocols. The lipid droplet quantification for ORO were measured with ImageJ software. Adipocyte size was quantified using perimeter tracings analyzed with ImageJ software. All the relative quantification data were normalized to the mean of the chow group.

### 2.19 Enzyme-linked immunosorbent assay (ELISA) assay

Serum insulin and leptin were analyzed using the ELISA kits (ALPCO Diagnostics, Salem, NH, USA) in accordance with the manufacturer's instructions.

### 2.20 Terminal deoxynucleotidyl transferase dUTP nick end labeling (TUNEL) assay

Apoptosis in mouse liver tissues was evaluated using the Alexa Fluor 640 TUNEL apoptosis detection kit (Cat# 40308) from Yeasen. The nuclei were counterstained with DAPI (5 μg/mL). To determine the average number of TUNEL-positive cells, ten different fields were analyzed under a IXM-4 fluorescence detection system (Molecular Devices) at 20× magnification.

### 2.21 Immunofluorescence and immunohistochemistry analysis

For the immunofluorescence analysis of SERCA2, cells or liver sections (5 μm thick) were first stained with a primary antibody specific to SERCA2 (1:100 dilution, Cat# A1097, ABclonal). A donkey anti-rabbit IgG conjugated with Alexa Fluor 594 (1:500, Cat# R37119, Thermo Fisher Scientific) served as the secondary antibody for fluorescence detection. Additionally, a DAPI solution at a concentration of 5 μg/mL was employed to counterstain the nuclei. Images were captured using the IXM-4 fluorescence detection system (Molecular Devices).

For the immunohistochemistry analysis of F4/80 antigen (F4/80), paraffin-embedded liver tissue sections were stained with an anti-F4/80 antibody (Cat# 70076, Cell Signaling Technology) at a dilution of 1:100. This was followed by incubation with an horseradish peroxidase (HRP)-linked secondary antibody. The stained sections were then visualized using a light microscope (CX23, Olympus, Tokyo, Japan).

### 2.22 Statistical analysis

All data are presented as means ± standard error of the mean (SEM) and were analyzed using PRISM 5 software (GraphPad Software, Inc). For comparisons between two groups, a Student's *t*-test was applied, while comparisons among multiple groups were conducted using one-way analysis of variance (ANOVA) with Bonferroni's post hoc test for further analysis. Statistical significance was established at *P* values less than 0.05.

## 3 Results

### 3.1 AT-II activates FXR and relieves ER stress in hepatocytes

**Fig. 1:**
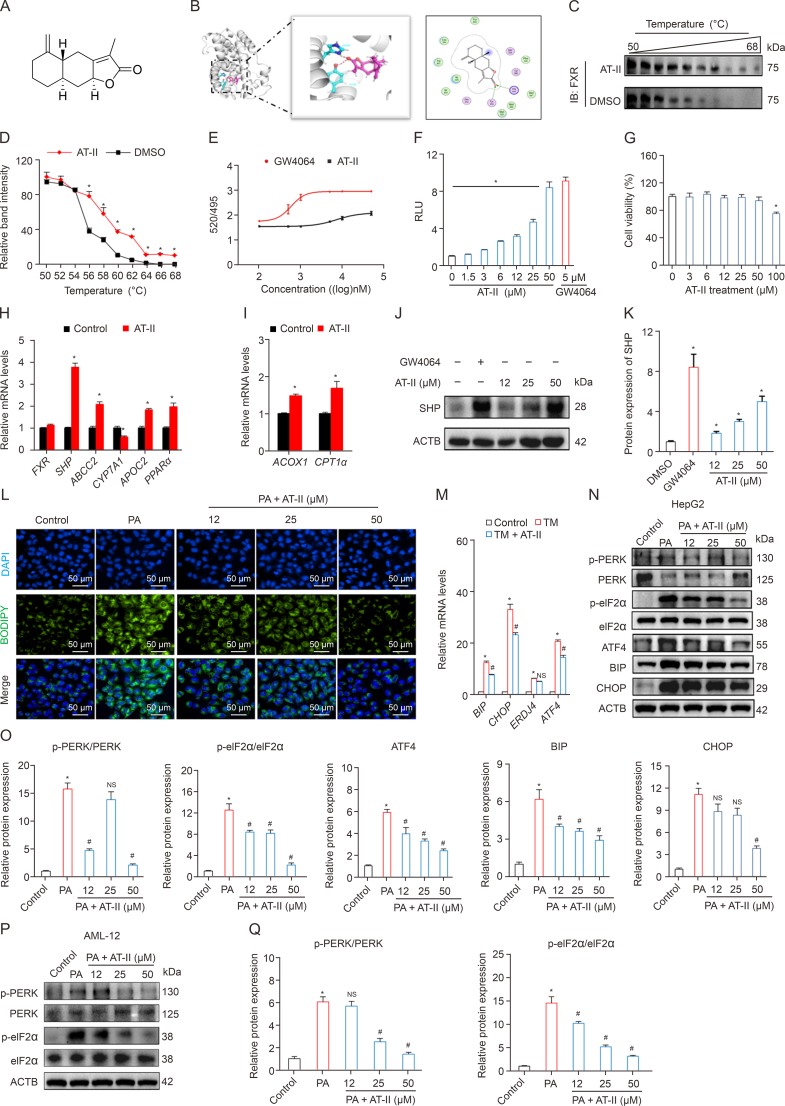
The potential role of atractylenolide II (AT-II) in regulating farnesoid X receptor (FXR) signaling and endoplasmic reticulum (ER) stress in hepatocytes. (A) An overview of AT-II's chemical structure. (B–E) The affinity of AT-II with FXR was evaluated using a molecular docking (B), cellular thermal shift assay (CETSA) analysis in HepG2 cells under temperature gradients (C, D), and a time-resolved fluorescence resonance energy transfer (TR-FRET) test for FXR (E). *n* = 3. The 293T cells were exposed to AT-II and GW4064 at specified doses for 24 h before harvesting, with dimethyl sulfoxide (DMSO) treatment as negative control. (F) Dual luciferase reporter gene assays of FXR transcriptional activity in 293T cells were analyzed, reporting relative light unit (RLU). *n* = 5. (G–K) The HepG2 cells were treated with AT-II or GW4064 for 24 h before harvesting, with DMSO treatment as negative control. (G) Cell counting kit 8 (CCK8) assessments. *n* = 5. (H, I) Relative messenger RNA (mRNA) expression of target genes in FXR (H), and peroxisome proliferator-activated receptor α (PPARα) (I) signaling were detected using quantitative real-time polymerase chain reaction (qRT-PCR). *n* = 5. (J, K) The small heterodimer partner (SHP) protein levels were detected by Western blotting (J), and quantified (K). *n* = 3. (L–O) HepG2 cells were treated with 2 μg/mL tunicamycin (TM) or 400 μM of palmitic acid (PA) with or without AT-II co-incubation for 24 h before harvesting. (L) Representative fluorescence images of boron-dipyrromethene (BODIPY) staining for intracellular lipid droplets observation. Image taken at ×400 magnification. (M) Relative mRNA levels of ER stress markers in DMSO or TM-treated HepG2 cells. *n* = 3. (N, O) Protein levels of unfolded protein response (UPR) signaling in PA-induced HepG2 model were detected (N), and quantified (O). *n* = 3. (P, Q) AML-12 cells were treated with PA (400 μM) with or without AT-II co-incubation for 24 h, and then subjected to Western blotting analysis of phosphorylation levels in protein kinase RNA-like ER kinase (PERK) and eukaryotic translation initiation factor 2 subunit α (eIF2α) (P), and quantified (Q). *n* = 3. The actin beta (ACTB) was used for data normalization in qRT-PCR and Western blotting results. Data were presented as the means ± standard error of the mean (SEM). ^∗^*P* < 0.05, compared to control group, ^#^*P* < 0.05, compared to TM or PA group, NS: no significance. IB: immunoblotting; DAPI: 4′,6-diamidino-2-phenylindole; ABCC2: ATP-binding cassette sub-family C member 2; CYP7A1: cytochrome p450 family 7 subfamily a member 1; APOC2: apolipoprotein C-II; ACOX1: acyl-CoA oxidase 1; CPT1α: carnitine palmitoyltransferase 1 α; BIP: glucose-regulated protein 78; ERDJ4: endoplasmic reticulum dnaJ heat shock protein 4; ATF4: activating transcription factor 4; CHOP: C/EBP Homologous Protein.

The specific roles of AT-II (Fig. 1A) in modulating FXR signaling are not yet fully understood. However, through molecular docking studies, we identified that AT-II might directly interact with Tyr358 and His444 within the binding pocket of FXR through hydrogen bonds, suggesting that AT-II could act as a ligand for FXR (Fig. 1B). This hypothesis was also supported by CETSA results, a heat stabilization assay used to validate ligand binding to target proteins. The assay revealed that FXR proteins in AT–II–treated cells exhibited increased thermal stability compared to control cells, with stabilization temperatures rising from 56 to 68 °C (Figs. 1C and D). In the FXR coactivator assay, AT-II activated TR-FRET signaling with a corresponding EC_50_ value of approximately 7.3 μM (Fig. 1E), further confirming the direct binding of AT-II to FXR. Next, in dual-luciferase reporter gene assays, AT-II showed a concentration-dependent activation of FXR transcriptional activity in the concentration range of 0–50 μM (Fig. 1F), and this concentration range of AT-II did not show significant cytotoxicity in hepatocytes (Fig. 1G). Moreover, AT-II significantly upregulated mRNA expression of FXR target genes such as *SHP*, *ABCC2*, *APOC2*, *PPARα*, *ACOX1*, and *CPT1α*, concomitant with the downregulation of *CYP7A1* expression in hepatocytes (Figs. 1H–K). Collectively, Our results revealed the agonistic properties of AT-II towards FXR.

To explore how AT-II modulates lipid metabolism in hepatocytes, we employed a PA-induced *in vitro* fatty liver model. Bodipy staining demonstrated that AT-II effectively inhibited excessive lipid accumulation caused by PA (Fig. 1L), suggesting a protective role against hepatic steatosis and ER stress. To further confirm AT-II's effect on reducing ER stress in hepatocytes, we utilized *in vitro* ER stress models with human and murine-derived hepatocytes. AT-II treatment significantly suppressed the upregulation of ER stress markers induced by TM with the exception of *ERDJ4* (Fig. 1M). Additionally, AT-II inhibited the phosphorylation of PERK and eIF2α and reduced the protein expression of ATF4, BIP, and CHOP in HepG2 cells (Figs. 1N and O). Furthermore, AT-II decreased the levels of p-PERK/PERK and p-eIF2α/eIF2α in AML-12 cells (Figs. 1P and Q). These findings indicate that AT-II can alleviate ER stress in hepatocytes by inhibiting the PERK/eIF2α-mediated UPR response.

### 3.2 AT-II activates SERCA2 through FXR agonism

**Fig. 2:**
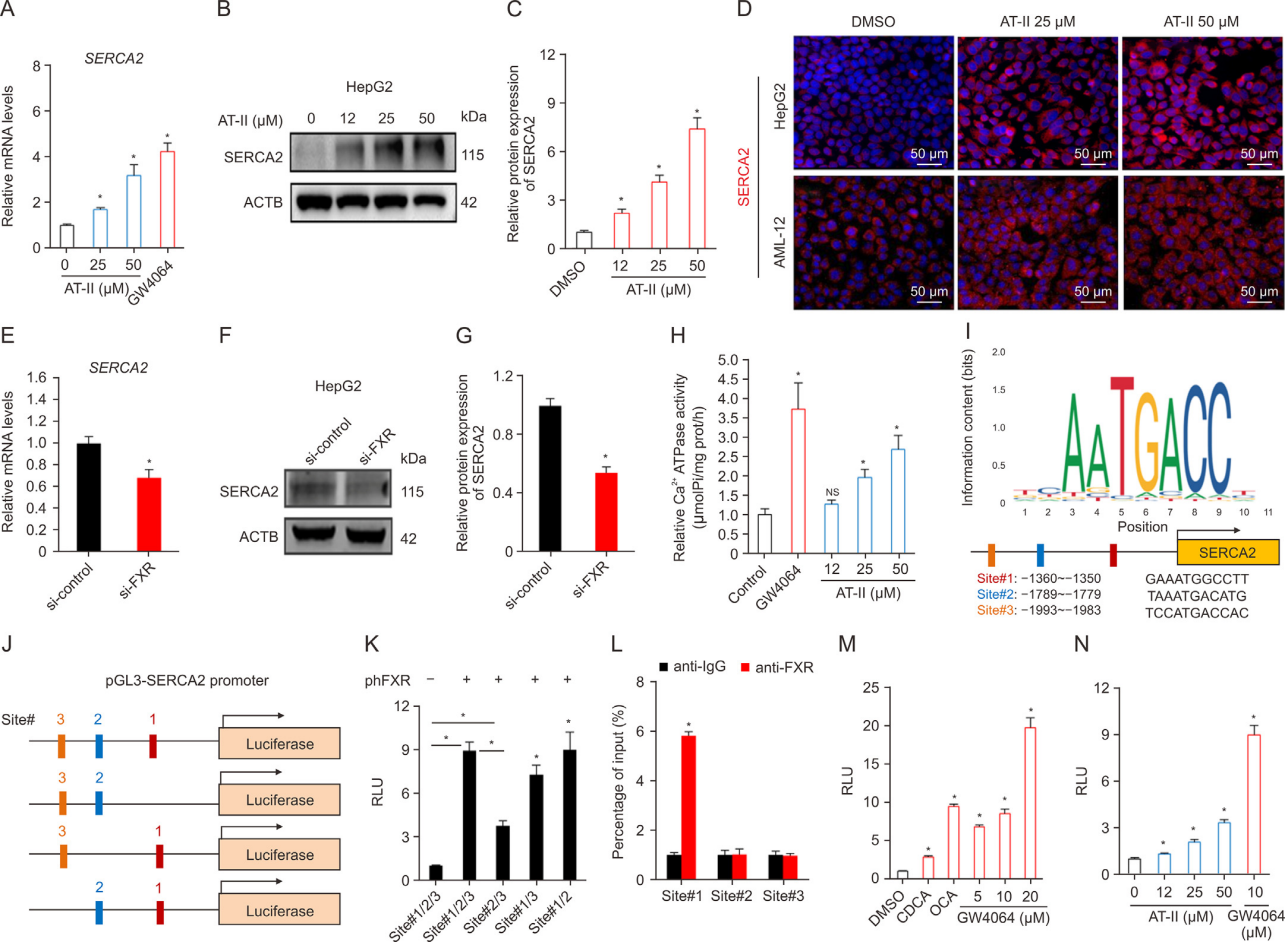
Atractylenolide II (AT-II) activates farnesoid X receptor (FXR)-sarco/endoplasmic reticulum Ca^2+^ ATPase 2 (SERCA2) axis in hepatocytes. (A–D) HepG2 or AML-12 cells were treated with AT-II or GW4064 (5 μM) for 24 h before harvesting, with dimethyl sulfoxide (DMSO) treatment as negative control. *n* = 3. (A) Relative messenger RNA (mRNA) expression of *SERCA2* in HepG2 cells. (B, C) Protein levels of SERCA2 in HepG2 cells were analyzed by Western blotting (B), and quantified (C). (D) Immunofluorescence detection of SERCA2 (red) in HepG2 or AML-12 cells. (E–G) HepG2 cells receiving a 48-h *FXR* siRNA transfection were subjected to the detection of relative mRNA (E), and protein (F, G) expression of SERCA2. *n* = 3. (H) HepG2 cells were treated with AT-II or GW4064 (5 μM) for 24 h before harvesting. Ca^2+^ ATPase activity was measured. *n* = 5. (I) Predicted FXR binding motifs in the −3000 SERCA2 promoter region. (J) An illustration of constructed pGL3-SERCA2 promoter plasmids and its mutants for luciferase reporter assays. (K) HepG2 cells were transfected with pGL3-SERCA2 promoter and its mutants, with or without hFXR overexpression. The activities of corresponding promoters were assessed via luciferase reporter assays. *n* = 5. (L) HepG2 cells were overexpressed with or without hFXR. Chromatin immunoprecipitation (ChIP) analysis of FXR binding to the SERCA2 promoter region in HepG2 cells were analyzed. *n* = 5. (M, N) HepG2 cells were over expressed with pGL3-SERCA2 and hFXR plasmids, and then treated with 0.1 % DMSO, 10 μM of chenodeoxycholic acid (CDCA), 10 μM of obeticholic acid (OCA), GW4064, and AT-II. pGL3-SERCA2 reporter analysis were conducted using resuling cells. *n* = 5. Data were indicated as the means ± standard error of the mean (SEM). ^∗^*P* < 0.05, vs. control group or si-control group, NS: no significance. RLU: relative light unit; IgG: immunoglobulin G.

Interestingly, treatment of HepG2 cells with AT-II or the FXR agonist GW4064 resulted in increased mRNA (Fig. 2A) and protein expression (Figs. 2B and C) of the ER stress inhibitory protein SERCA2. This effect was further confirmed by immunofluorescence analysis, which demonstrated that AT-II significantly enhanced in situ SERCA2 protein expression in both HepG2 and AML-12 cells (Fig. 2D). In contrast, knockdown of *SERCA2* led to a reduction in its expression in HepG2 cells (Figs. 2E–G). Given that SERCA2 is a crucial intracellular Ca^2+^ ATPase, we assessed the Ca^2+^ ATPase activity in hepatocytes and found that treatment with AT-II and GW4064 significantly increased Ca^2+^ ATPase activity in HepG2 cells (Fig. 2H). These findings suggest that FXR activation promotes SERCA2 expression and activity in hepatocytes.

To further investigate whether FXR can transcriptionally regulate SERCA2 expression, we utilized the JASPAR database, which predicted three potential FXR binding sites within the SERCA2 promoter (Fig. 2I). We then constructed four reporter gene plasmids, each containing either the full or partial sequences of the SERCA2 promoter as illustrated in Fig. 2J. These plasmids were transfected into HepG2 cells, either with or without FXR overexpression plasmids, for SERCA2 promoter luciferase analysis. The results showed that FXR overexpression significantly increased SERCA2 promoter luciferase activity in conditions that included Site#1, Site#2, and Site#3, or when Site#1, Site#2, or Site#3 were deleted individually (Fig. 2K). However, deletion of Site#1 notably reduced this induction by FXR overexpression, while deletion of the other two sites had no significant effect (Fig. 2K). This suggests that FXR is most likely to bind to the sequence at site#1. This finding was further supported by ChIP assay results (Fig. 2L), which showed increased FXR occupancy at Site#1 in the SERCA2 promoter with FXR overexpression. Consistent with these findings, *in vitro* pharmacological studies demonstrated that various FXR agonists, including CDCA, OCA, GW4064, and AT-II, significantly enhanced SERCA2 promoter activity (Figs. 2M and N). These results indicate that AT-II promotes SERCA2 transcription through FXR activation.

### 3.3 AT-II restores lipid and glucose metabolism in obese mice

**Fig. 3:**
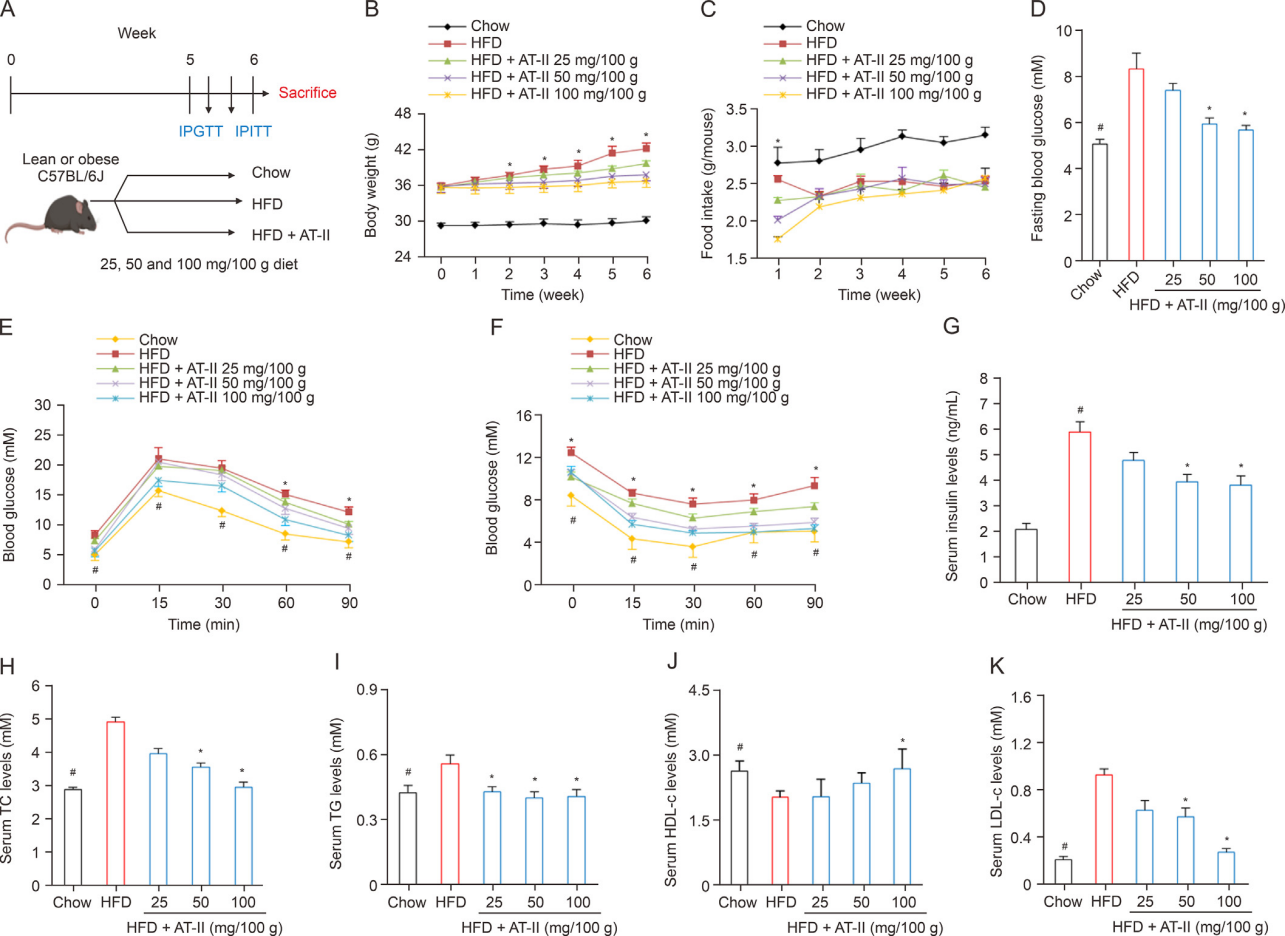
Atractylenolide II (AT-II) inhibits bodyweight gain and improves metabolic dysfunctions in diet-induced obese (DIO) mice. (A) Schematic diagram of the DIO experiment design. (B–K) Over a six-week period, obese male C57BL/6 mice were fed high-fat diets (HFD) or HFD containing AT-II (25, 50, or 100 mg/100 g diet). *n* = 7. (B) Weight gain curve. *n* = 7 per group. (C) Food intake record. *n* = 7. (D) Blood glucose level at fasting. *n* = 7. (E) Intraperitoneal glucose tolerance tests (IPGTT) in all groups of mice. *n* = 7. (F) Intraperitoneal insulin tolerance tests (IPITT) in all groups of mice. *n* = 7. (G) Enzyme-linked immunosorbent assay (ELISA) for serum insulin levels. *n* = 6. (H–K) Biochemical tests for serum lipids levels of total cholesterol (TC) (H), triglyceride (TG) (I), high-density lipoprotein cholesterol (HDL-c) (J), and low-density lipoprotein cholesterol (LDL-c) (K). *n* = 7. Data were analyzed as the means ± standard error of the mean (SEM). ^∗^*P* < 0.05, HFD vs. Chow; ^#^*P* < 0.05, HFD vs. HFD + AT-II.

Subsequent to the DIO mouse model study (Fig. 3A), our assessment revealed that persistent HFD feeding significantly increased weight gain in mice, a trend that was effectively interrupted by AT-II treatment (Fig. 3B). During the first week, AT-II initially reduced food consumption, though this effect was not sustained (Fig. 3C). Subsequent serum biochemical assays indicated that HFD feeding led to abnormal fasting blood glucose levels (Fig. 3D), glucose intolerance (Fig. 3E), decreased insulin sensitivity (Fig. 3F), and elevated insulin levels (Fig. 3G) compared to chow-fed controls. Notably, these adverse effects were significantly improved with AT-II treatment (Figs. 3D–G). Serum lipid assessments also revealed increased levels of TG, TC, and LDL-c, along with decreased HDL-c in HFD-fed mice, all of which were substantially reversed by AT-II treatment (Figs. 3H–K). However, AT-II treatment did not affect fecal TG excretion in mice (Fig. S1). These findings suggest that AT-II effectively reduces body weight gain and ameliorates disturbances in IR and glucose-lipid metabolism in DIO mice.

### 3.4 AT-II promotes energy production in mice

**Fig. 4:**
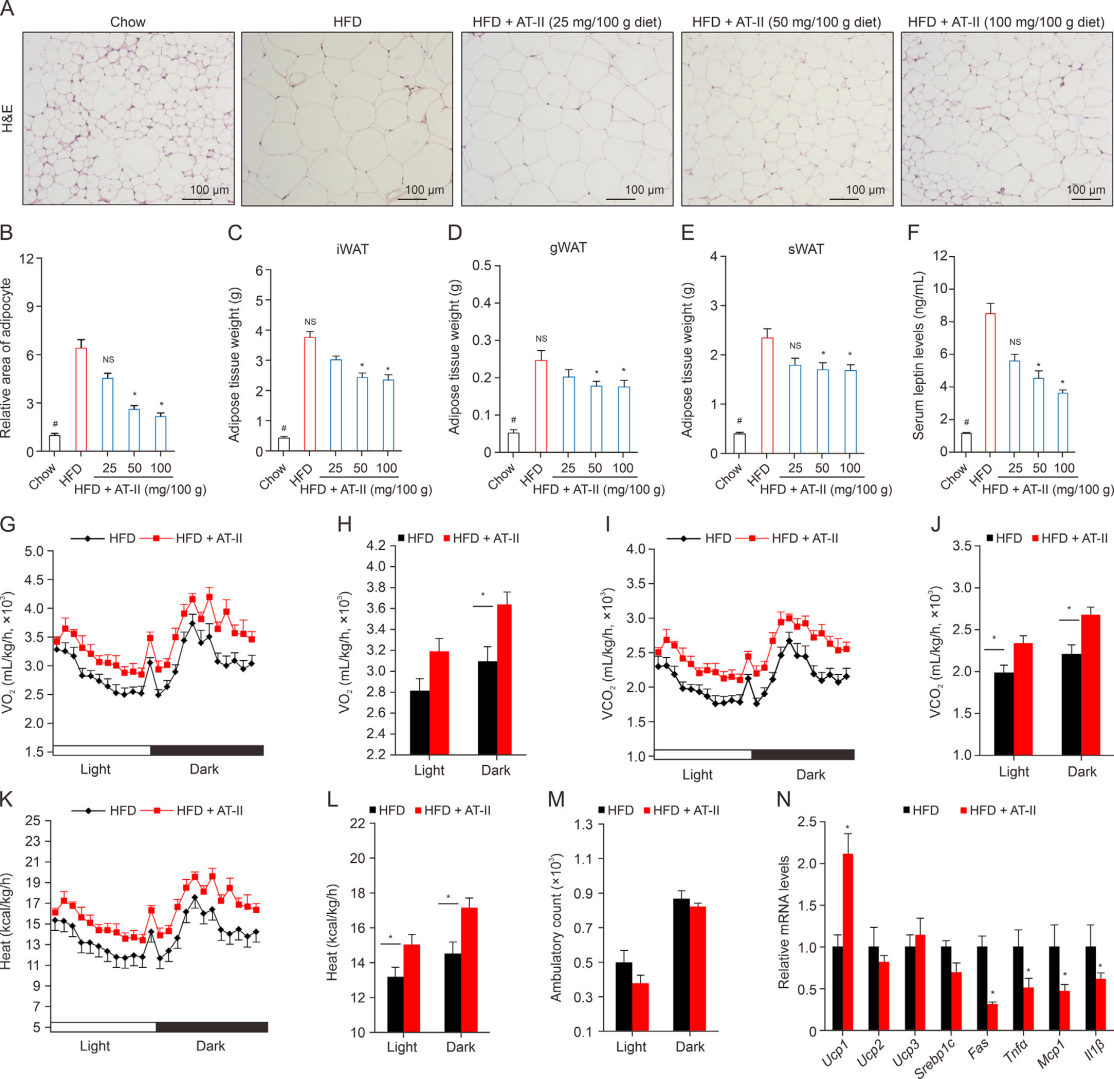
Atractylenolide II (AT-II) reduces adipose tissue size and facilitates energy expenditure in diet-induced obese (DIO) mice. (A–E) Obese male C57BL/6 mice were fed high-fat diets (HFD) or HFD containing AT-II for six weeks before harvesting. *n* = 7. The white adipose tissues (WAT) were collected for hematoxylin and eosin (H&E) staining (A), calculation of the relative area of WAT based on cell diameters (B), weighing of inguinal WAT (iWAT) (C), gonadal WAT (gWAT) (D), and subcutaneous WAT (sWAT) (E). (F) Serum leptin levels by enzyme-linked immunosorbent assay (ELISA). *n* = 7. (G–M) Twenty-four-hour metabolic parameters in HFD and HFD + AT-II (100 mg/100 g diet) group mice were recorded. *n* = 5. (G, H) Oxygen consumption (VO_2_). (I, J) Carbon dioxide production (VCO_2_). (K, L) Heat production. (M) Locomotor activity. (N) Relative gene expression analysis in WAT. *n* = 5. All data were shown as the means ± standard error of the mean (SEM). ^∗^*P* < 0.05, HFD vs. Chow; ^*#*^*P* < 0.05, HFD vs. HFD + AT-II, NS: no significance. UCP: uncoupling protein; SREBP1c: sterol regulatory element-binding protein 1c; FAS: fatty acid synthase; TNFα: tumor necrosis factor α; MCP1: monocyte chemoattractant protein-1; IL1β: interleukin 1 β.

To determine whether AT-II's inhibition of weight gain in DIO mice is associated with reduced adipose tissue, we performed in situ staining of white adipose tissues (WAT) using H&E. The adipocyte size in HFD-fed mice was significantly larger compared to chow-fed controls and AT–II–treated mice (Figs. 4A and B). This observation was further supported by weight measurements of inguinal, gonadal, and subcutaneous WAT, which were notably lower in AT–II–treated mice (Figs. 4C–E). Additionally, AT-II significantly reduced the elevated serum leptin levels induced by HFD feeding (Fig. 4F), further confirming its anti-obesity effects in DIO mice. To explore whether AT-II enhances energy expenditure, mice from the HFD group and the AT-II treatment group (100 mg/100 g diet) were placed in metabolic cages. Compared to the HFD group, AT–II–treated mice exhibited higher rates of VO_2_ (Figs. 4G and H), VCO_2_ (Figs. 4I and J), respiratory exchange ratio (Fig. S2), and heat production (Figs. 4K and L), with no significant difference in ambulatory activity (Fig. 4M). These findings suggest that AT-II's anti-obesity effects are primarily due to increased energy expenditure. Furthermore, AT-II significantly upregulated the mRNA expression of the thermogenesis gene *Ucp1* in WAT while downregulating the mRNA levels of the lipogenic gene *Fas* and pro-inflammatory genes *Tnfα*, *Mcp1*, and *Il1β* compared to HFD controls (Fig. 4N). This indicates that AT-II promotes energy expenditure and reduces inflammation in adipose tissue.

### 3.5 AT-II alleviates steatotic liver in DIO mice

**Fig. 5:**
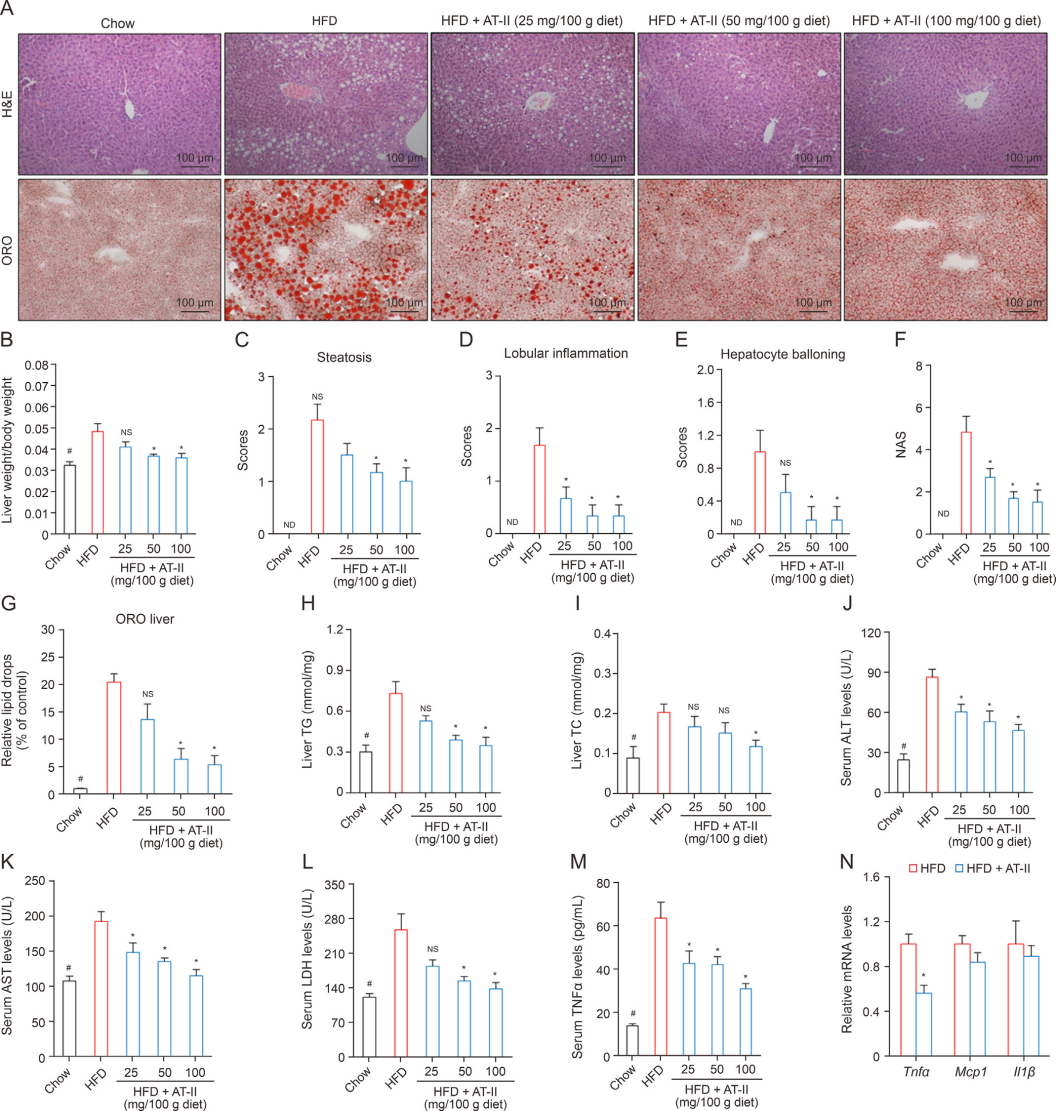
Atractylenolide II (AT-II) ameliorates fatty liver in diet-induced obese (DIO) mice. (A–G) Obese male C57BL/6 mice were fed high-fat diets (HFD) or HFD containing AT-II for six weeks before harvesting. The liver tissues were collected for hepatic hematoxylin and eosin (H&E) and Oil-Red O (ORO) staining (A). Calculation of the liver weight to body weight ratio (B), pathological scoring for steatosis (C), lobular inflammation (D), hepatocyte ballooning (E), and non-alcoholic fatty liver disease activity score (NAS) (F), and relative ORO quantification (G). *n* = 6. (H, I) The mouse liver tissues from DIO experiments were subjected to determination of triglyceride (TG) (H) and total cholesterol (TC) (I) content. *n* = 7. (J–L) The mouse serum from DIO experiments were collected for biochemical assays of alanine aminotransferase (ALT) (J), aspartate aminotransferase (AST) (K), and lactic dehydrogenase (LDH) (L). *n* = 7. (M) Serum tumor necrosis factor α (TNFα) levels were determined by enzyme-linked immunosorbent assay (ELISA). *n* = 7. (N) Hepatic pro-inflammatory genes were assessed by quantitative real-time polymerase chain reaction (qRT-PCR). *n* = 5. All data were analyzed as the means ± standard error of the mean (SEM). ∗*P* < 0.05, HFD vs. Chow; ^#^*P* < 0.05, HFD vs. HFD + AT-II, NS: no significance, ND: not detected.

We then evaluated the lipid-lowering and anti-inflammatory effects of AT-II on the livers of DIO mice. Analysis of the liver weight to body weight ratio and liver pathology revealed that HFD-induced fatty liver, characterized by increased hepatic steatosis, inflammation, hepatocyte ballooning, and elevated liver weight, was significantly mitigated by AT-II treatment (Figs. 5A and B). These findings were corroborated by improved pathological scores of the liver (Figs. 5C–F) and ORO staining in the liver (Figs. 5A and G). Further examination of hepatic TG and TC levels showed that HFD-fed mice had significantly higher levels compared to the chow-fed group, but AT-II treatment markedly reduced these levels in DIO mice (Figs. 5H and I). To assess the protective effects of AT-II against liver damage caused by HFD, we measured serum levels of ALT, AST, and LDH. HFD-fed mice exhibited significantly higher levels of these liver enzymes compared to chow controls, while AT-II treatment resulted in significantly lower levels of ALT, AST, and LDH compared to the HFD group (Figs. 5J–L). Moreover, AT-II treatment led to a reduction in the concentrations of the pro-inflammatory factor TNFα, both in serum (Fig. 5M) and hepatic mRNA levels (Fig. 5N), indicating decreased liver inflammation. These results suggest that AT-II effectively improves NAFLD in DIO mice by reducing hepatic steatosis, inflammation, and liver damage.

### 3.6 AT-II counteracts MCD-induced NASH in mice

**Fig. 6:**
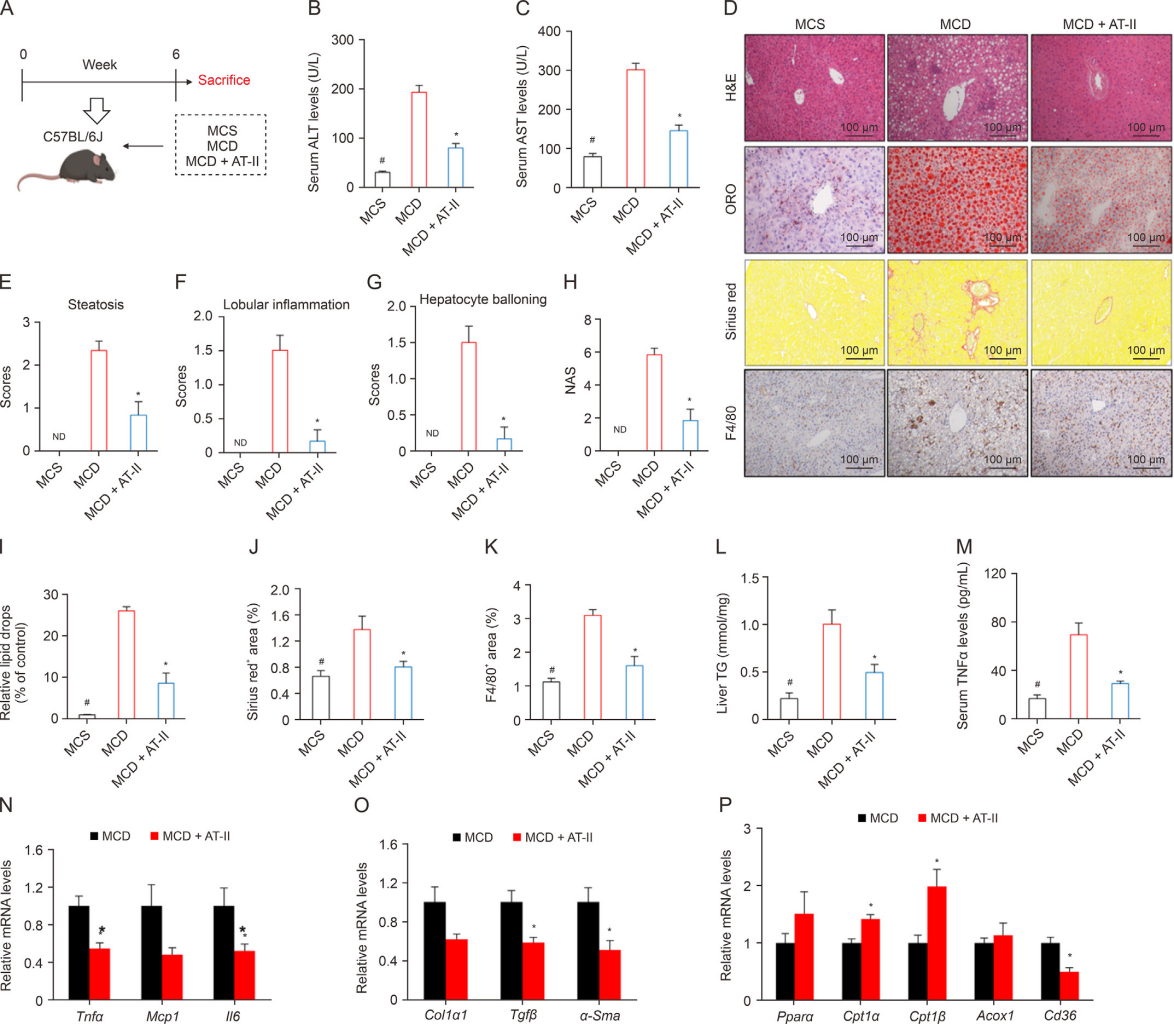
Atractylenolide II (AT-II) protects mice from methionine and choline-deficient l-amino acid diets (MCD)-induced nonalcoholic fatty liver disease (NASH). (A) Schematic of the MCD inducement experiment. (B, C) Methionine and choline-sufficient l-amino acid diets (MCS) or MCD mixed with AT-II (100 mg/100 g diet) was fed to C57BL/6 mice for six weeks. The serum were collected for alanine aminotransferase (ALT) (B), and aspartate aminotransferase (AST) (C) examination. *n* = 6. (D–L) The liver tissues from MCD experiments were harvested. (D) Staining with hematoxylin and eosin (H&E), Oil-Red O (ORO), and Sirius red, and F4/80 antigen (F4/80)immunohistochemistry. (E–H) Hepatic scores of steatosis (E), lobular inflammation (F), hepatocyte ballooning (G), and non-alcoholic fatty liver disease activity score (NAS) (H). *n* = 6. (I) Relative ORO quantification in the livers. *n* = 6. (J) Quantification of hepatic Sirius red^+^ area. *n* = 6. (K) Quantification of hepatic F4/80^+^ area. *n* = 6. (L) Hepatic triglyceride (TG) levels. *n* = 6. (M) Serum tumor necrosis factor α (TNFα) levels were determined by enzyme-linked immunosorbent assay (ELISA). *n* = 6. (N–P) Relative mNRA levels of genes in the liver were analyzed by quantitative real-time polymerase chain reaction (qRT-PCR). *n* = 5. All data were shown as the means ± standard error of the mean (SEM). ^#^*P* < 0.05, MCD vs. MCS; ∗*P*< 0.05, MCD vs. MCD + AT-II group. ND: not detected; MCS: methionine and choline-sufficient l-amino acid diets; MCP1: monocyte chemoattractant protein-1; IL6: interleukin 6; COL1α1: collagen type I α1 chain; TGFβ: transforming growth factor β; α-SMA: α-smooth muscle actin; PPARα: peroxisome proliferator-activated receptor α; CPT1: carnitine palmitoyltransferase 1; ACOX1: acyl-CoA oxidase 1; CD36: cluster of differentiation 36.

To validate the protective effects of AT-II against NASH, we employed a mouse model of NASH induced by MCD feeding (Fig. 6A). After 6 weeks of AT-II treatment, we observed a significant reversal of the MCD-induced elevations in serum ALT and AST levels (Figs. 6B and C). Liver pathology analysis showed that MCD feeding resulted in pronounced steatosis, inflammatory cell infiltration, and fibrosis, all of which were significantly alleviated by AT-II treatment (Figs. 6D–J). Immunohistochemical analysis using the macrophage marker F4/80 further demonstrated reduced macrophage presence in liver tissue following AT-II treatment (Figs. 6D and K), confirming its anti-hepatitis effects. Moreover, quantitative measurement of hepatic TG confirmed that AT-II effectively reduced liver fat content in NASH mice (Fig. 6L). Additionally, the MCD group showed a significant increase in serum TNFα compared to the MCS control group, a trend that was notably mitigated by AT-II treatment (Fig. 6M). AT-II also decreased the mRNA expression of pro-inflammatory genes (*Tnfα* and *Il6*), pro-fibrotic genes (*Tgfβ* and *α-Sma*), and the lipid transporter *Cd36*, while enhancing the expression of fatty acid β-oxidation (FAO) genes (*Cpt1α/β*) (Figs. 6N–P). These findings suggest that AT-II exerts its anti-NASH effects in MCD-fed mice through multiple mechanisms, including reducing inflammation, fibrosis, and liver fat content, and promoting FAO.

### 3.7 AT-II alleviates fatty liver in mice under chemical condition inducing ER stress

**Fig. 7:**
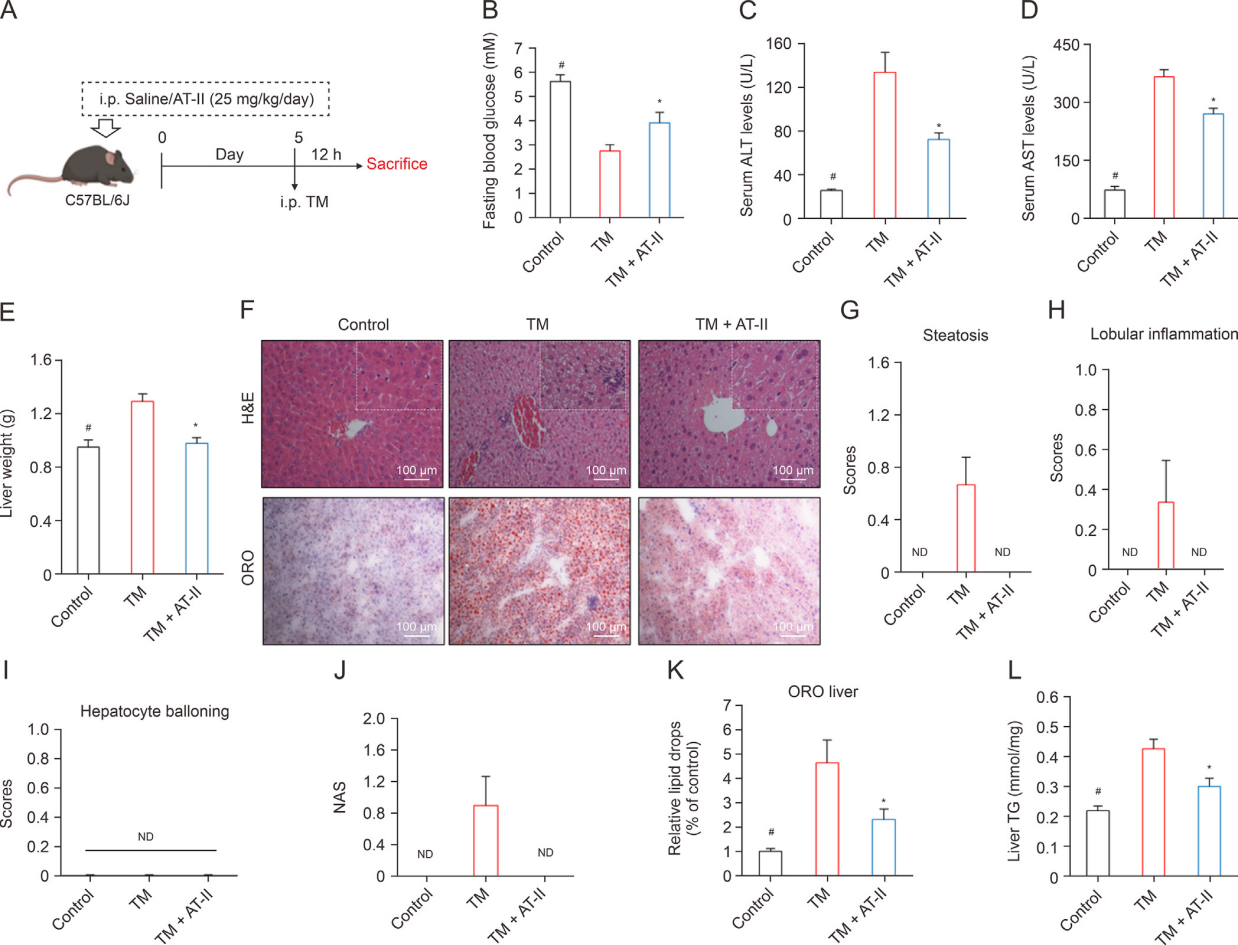
Atractylenolide II (AT-II) improves tunicamycin (TM)-induced steatosis in mice. (A) A schematic diagram of TM animal model design. Male C57BL/6 mice were treated AT-II (i.p. 25 mg/kg) for 7 days followed by a single administration of TM (i.p. 1 mg/kg). (B) Fasting blood glucose. (C, D) Tests for serum alanine aminotransferase (ALT) (C), and aspartate aminotransferase (AST) (D). (E) Liver weight measurement. (F–L) Liver tissues were collected for staining with hematoxylin and eosin (H&E) and Oil-Red O (ORO) (F). Evaluation of steatosis (G), lobular inflammation (H), hepatocyte ballooning (I), and non-alcoholic fatty liver disease activity score (NAS) (J), and ORO quantification (K). (L) Hepatic triglyceride (TG) determination. All data were presented as the means ± standard error of the mean (SEM). *n* = 6. ^#^*P* < 0.05, TM vs. Control; ∗*P* < 0.05, TM vs. TM + AT-II group. ND: not detected.

TM, a commonly used chemical inducer of ER stress, was employed to create animal models of fatty liver and metabolic abnormalities. We then investigated the effectiveness of AT-II in counteracting TM-induced damage (Fig. 7A) to establish a direct link between AT-II's beneficial effects in managing NAFLD and its ability to resist ER stress. Acute TM injection almost induced hypoglycemia in normal male C57/BL6 mice, a condition that was significantly prevented by AT-II intervention (Fig. 7B). Additionally, TM treatment resulted in a significant increase in serum ALT and AST levels compared to the control group, whereas AT-II-treated mice showed lower ALT and AST levels than the TM group (Figs. 7C and D), indicating that TM caused hepatic injury that was mitigated by AT-II. Furthermore, TM-injected mice exhibited higher liver weights compared to controls, while this increase was not observed in the AT-II treatment group (Fig. 7E). Liver histopathological analysis and scoring using H&E and ORO staining demonstrated that TM modeling led to excessive fat accumulation in the liver, a condition largely prevented by AT-II treatment (Figs. 7F–K), further suggesting AT-II's ability to inhibit fatty liver development. This was corroborated by the assessment of liver TG content (Fig. 7L). These findings underscore the potential of AT-II in improving ER stress-associated fatty liver in mice.

### 3.8 AT-II promotes hepatic FXR-SERCA2 signaling

**Fig. 8:**
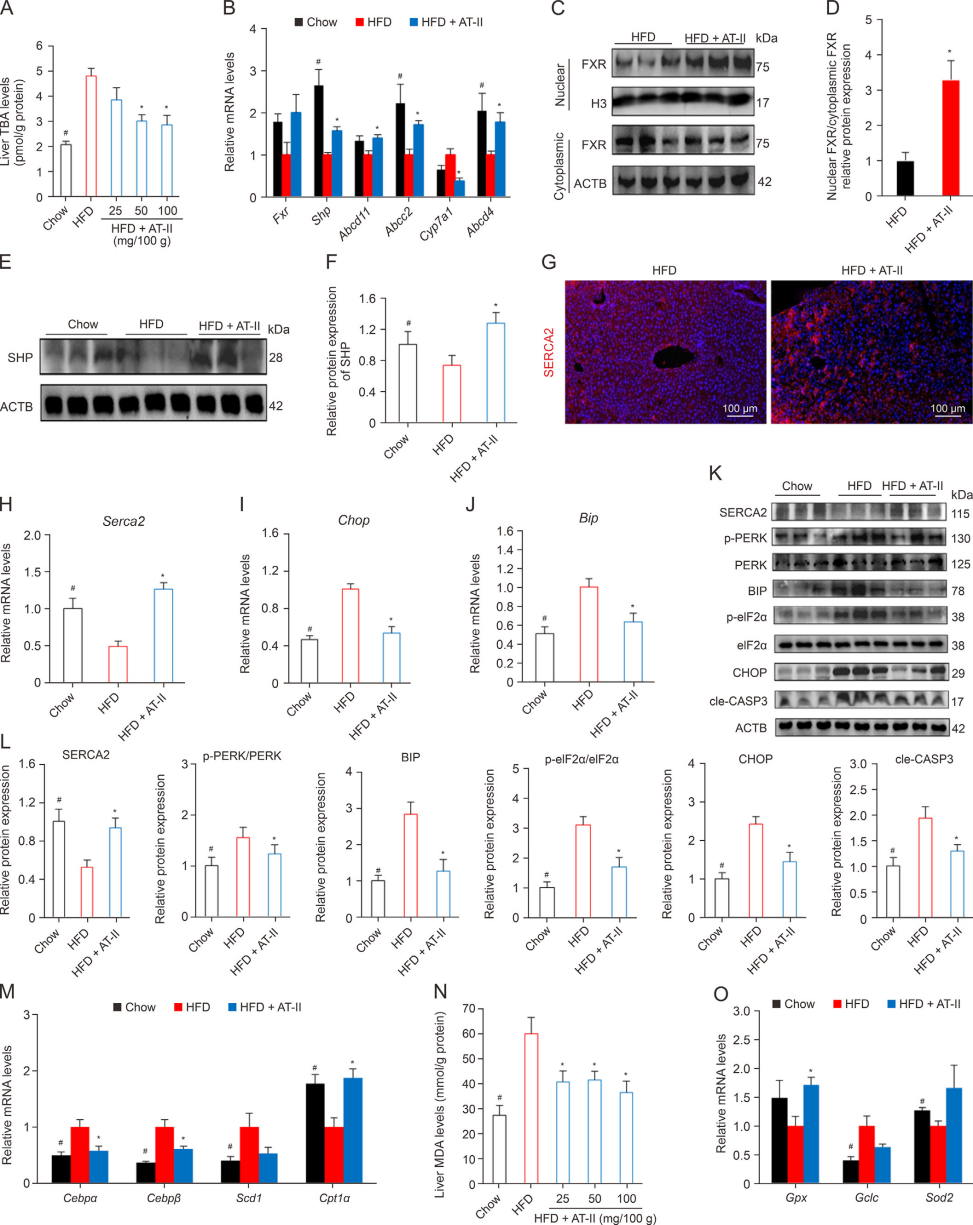
Activation of the farnesoid X receptor (FXR)-sarco/endoplasmic reticulum Ca^2+^ ATPase 2 (SERCA2) axis in hepatic cells by atractylenolide II (AT-II) in diet-induced obese (DIO) mice. A high-fat diets (HFD) with or without AT-II (100 mg/100 g diet) was administered to obese male C57BL/6 mice for six weeks. (A) Hepatic total bile acid (TBA) levels. *n* = 7. (B) Hepatic expression of FXR signaling genes were measured by quantitative real-time polymerase chain reaction (qRT-PCR). *n* = 5. (C, D) Nuclear translocation of liver FXR was analyzed by Western blotting (C), and quantified (D). *n* = 6. Histone H3 (H3) and actin beta (ACTB) were used as internal references for nuclear and cytoplasmic protein expression, respectively. (E, F) Hepatic small heterodimer partner (SHP) protein levels were imaged by Western blotting (E), and quantified (F). *n* = 6. (G) Immunofluorescence detection of SERCA2 in the liver. (H–J) Hepatic messenger RNA (mRNA) levels of *Serca2* (H), C/EBP homologous protein (*Chop*) (I), and glucose-regulated protein 78 (*Bip*) were detected by quantitative real-time polymerase chain reaction (qRT-PCR). *n* = 5. (K, L) Protein expression of heptic SERCA2 and endoplasmic reticulum (ER) stress signaling were detected by Western blotting (K), and quantified (L). *n* = 6. (M) Hepatic lipid metabolism gene expression. *n* = 5. (N) Hepatic total malondialdehyde (MDA) levels. *n* = 7. (O) Relative mRNA expression of oxidative stress-related genes in the liver. *n* = 5. All data were indicated as the means ± standard error of the mean (SEM). ^∗^*P* < 0.05, HFD vs. Chow; ^#^*P* < 0.05, HFD vs. HFD + AT-II. ABCC2: ATP-binding cassette sub-family C member 2; ABCB11: ATP-binding cassette sub-family B member 11; ABCB4: ATP-binding cassette sub-family B member 4; CYP7A1: cytochrome p450 family 7 subfamily a member 1; cle-CASP3: cleaved Caspase-3; CPT1α: carnitine palmitoyltransferase 1 α; CEBP: CCAAT/enhancer binding protein; SCD1: stearoyl-CoA desaturase 1; GPX: glutathione peroxidase; GCLC: glutamate-cysteine ligase catalytic subunit; SOD2: superoxide dismutase 2.

We next examined the activation of FXR-SERCA2 signaling in the liver of mice treated with AT-II (100 mg/100 g diet). FXR activation is known to inhibit TBA synthesis [17]. Our analysis of hepatic TBA content revealed that HFD-fed mice had higher levels of hepatic TBA compared to chow-fed controls, whereas the AT-II-treated group exhibited significantly lower hepatic TBA levels than the HFD controls (Fig. 8A). Further experiments demonstrated that AT-II increased hepatic *Fxr* mRNA expression in HFD-fed mice (Fig. 8B). AT-II also promoted the expression of key FXR target genes, including *Shp*, *Abcb11*, *Abcb4*, and *Abcc2*, while downregulating the expression of the bile acid synthase *Cyp7a1* in the livers of HFD-fed mice (Fig. 8B). Subsequently, we found that AT-II treatment promoted hepatic FXR nuclear translocation in DIO mice (Figs. 8C and D). Additionally, AT-II upregulated the hepatic protein expression of SHP, an typical FXR downstream gene, in HFD-fed mice (Figs. 8E and F). These findings indicate that AT-II activates FXR signaling in the liver. Moreover, AT-II significantly increased the *in situ* expression of hepatic SERCA2 protein in DIO mice (Fig. 8G). Meanwhile, AT-II markedly upregulated *Serca2* mRNA levels (Fig. 8H) while decreasing mRNA levels of the ER stress markers *Bip* and *Chop* in the livers of HFD-fed mice (Figs. 8I and J).

We also found that HFD feeding significantly reduced SERCA2 protein levels (Figs. 8K and L) while elevating the expression of ER stress markers BIP and CHOP (Figs. 8K and L), and led to hyperphosphorylation of PERK and eIF2α in mice (Figs. 8K and L), effects that were mitigated by AT-II treatment (Figs. 8K and L). The increase in cleaved caspase-3 (cle-CASP3), an indicator of apoptosis, was also reversed by AT-II (Figs. 8K and L). Furthermore, compared to the HFD group, AT-II-treated mice exhibited lower expression levels of lipid synthesis genes *Cebpα/β* and *Scd1*, and significantly higher expression of the fatty acid FAO gene *Cpt1α*, bringing their gene expression profile closer to that of chow-fed control mice (Fig. 8M). Lastly, we observed that AT-II significantly reduced hepatic MDA content (Fig. 8N) and increased the mRNA expression of the antioxidant gene *Gpx* (Fig. 8O), further underscoring AT-II's inhibitory effect on hepatic ER stress. Collectively, these findings suggest that the activation of FXR-SERCA2 signaling plays a key role in the protective effects of AT-II against hepatic ER stress and fatty liver.

### 3.9 Hepatic Serca2 knock down impedes the anti-NAFLD action of AT-II

**Fig. 9:**
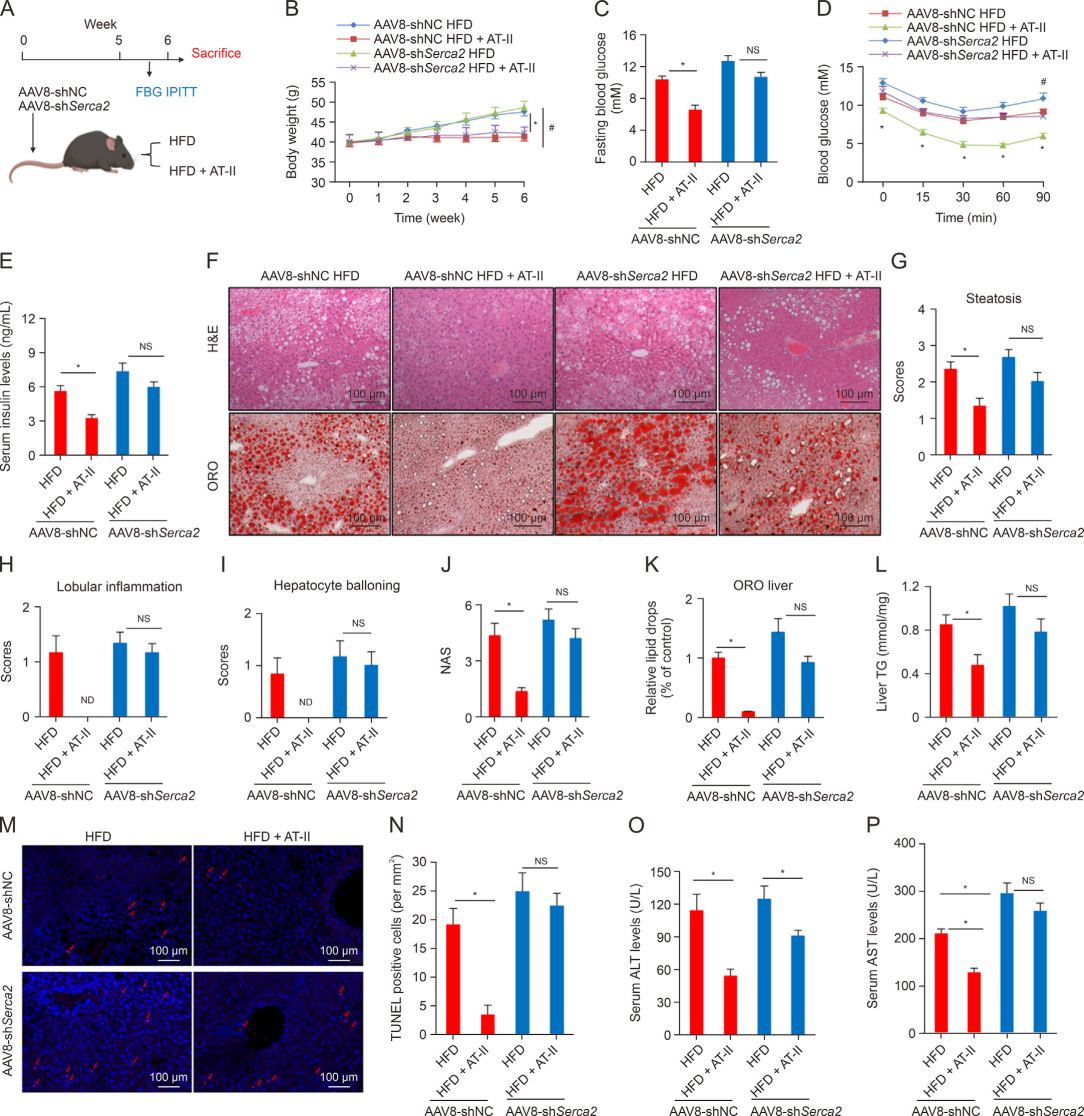
Adeno-associated virus 8 (AAV8)-mediated sarco/endoplasmic reticulum Ca^2+^ ATPase 2 (*Serca2*) silencing in the liver diminishes the efficacy of atractylenolide II (AT-II) against insulin resistance and fatty liver in diet-induced obese (DIO) mice. (A) Schematic of the animal model design. (B–E) Obese male C57BL/6 mice received tail vein injections of AAV8-shNC or AAV8-sh*Serca2* and were then fed a high-fat diets (HFD) with or without AT-II (100 mg/100 g diet) for six weeks. *n* = 6. (B) Body weight gain. (C) Fasting blood glucose. (D) Intraperitoneal insulin tolerance test (IPITT). (E) Enzyme-linked immunosorbent assay (ELISA) measurement of serum insulin levels. (F–K) The liver tissues from mice with AAV8 interference were subjected to hematoxylin and eosin (H&E) and Oil-Red O (ORO) staining (F), scoring of steatosis (G), lobular inflammation (H), hepatocyte ballooning (I), and non-alcoholic fatty liver disease activity score (NAS) (J), and ORO quantification (K). *n* = 6. (L) Total liver triglyceride (TG) content. *n* = 6. (M, N) Apoptosis in the liver tissues was analyzed by terminal deoxynucleotidyl transferase dUTP nick end labeling (TUNEL) (M), and the TUNEL positive cells were quantified (N). *n* = 6. (O, P) The serum from mice with AAV8 interference were subjected to analysis of alanine aminotransferase (ALT) (O), and aspartate aminotransferase (AST) (P). *n* = 6. All data were shown as the means ± standard error of the mean (SEM). ^∗^*P* < 0.05, AAV8-shNC HFD vs. AAV8-shNC HFD + AT-II; ^#^*P* < 0.05, AAV8-sh*Serca2* HFD vs. AAV8-sh*Serca2* HFD + AT-II; NS: no significance. ND: not detected; NC: negative control; FBG: fasting blood glucose.

To determine whether hepatic SERCA2 mediates the beneficial effects of AT-II in combating fatty liver, we performed hepatic *Serca2* knockdown in DIO mice by injecting the AAV8 virus via the tail vein (Fig. 9A). After 6 weeks of AT-II treatment (100 mg/100 g diet), we found that hepatic *Serca2* knockdown did not alter the weight loss induced by AT-II (Fig. 9B). However, notably, compared to the AAV8-shNC control group, the AAV8-sh*Serca2* injection negated AT-II's ability to lower fasting blood glucose in DIO mice (Fig. 9C). Additionally, under empty AAV8 control conditions, AT-II effectively enhanced insulin sensitivity (Fig. 9D) and reduced serum insulin levels (Fig. 9E) in obese mice, but these effects were significantly diminished by hepatic *Serca2* knockdown. These findings suggest that hepatic SERCA2 is crucial for AT-II's glycemic-lowering and insulin-sensitizing effects. Further pathological analysis of the liver showed that AT-II treatment significantly improved NAFLD in mice injected with the empty control virus, while hepatic *Serca2* knockdown markedly impaired AT-II's protective effect in the liver (Figs. 9F–K). This was further confirmed by measuring hepatic TG content (Fig. 9L). In further investigations, TUNEL analysis of liver tissues from DIO mice in the AAV8 viral interference trial showed a significant reduction in TUNEL-positive cells with AT-II treatment in mice injected with AAV8-shNC (Figs. 9M and N). However, this effect was absent in mice receiving AAV8-sh*Serca2* (Figs. 9M and N). Additionally, the ability of AT-II to reduce serum ALT levels was significantly impaired by AAV8-sh*Serca2* interference (Fig. 9O), and its effect on lowering serum AST levels was also lost (Fig. 9P). In fact, mice with AAV8-sh*Serca2* exhibited higher serum AST levels than those in the AAV8-shNC HFD group (Fig. 9P). These findings indicate that inhibiting hepatic SERCA2 exacerbates liver injury and negates the hepatoprotective effects of AT-II in HFD-fed obese mice. Collectively, these results demonstrate that the beneficial effects of AT-II on fatty liver and IR in mice require the involvement of hepatic SERCA2.

### 3.10 Dephosphorylation of eIF2α by activation of FXR-SERCA2 axis is involved in AT-II's anti-NAFLD

**Fig. 10:**
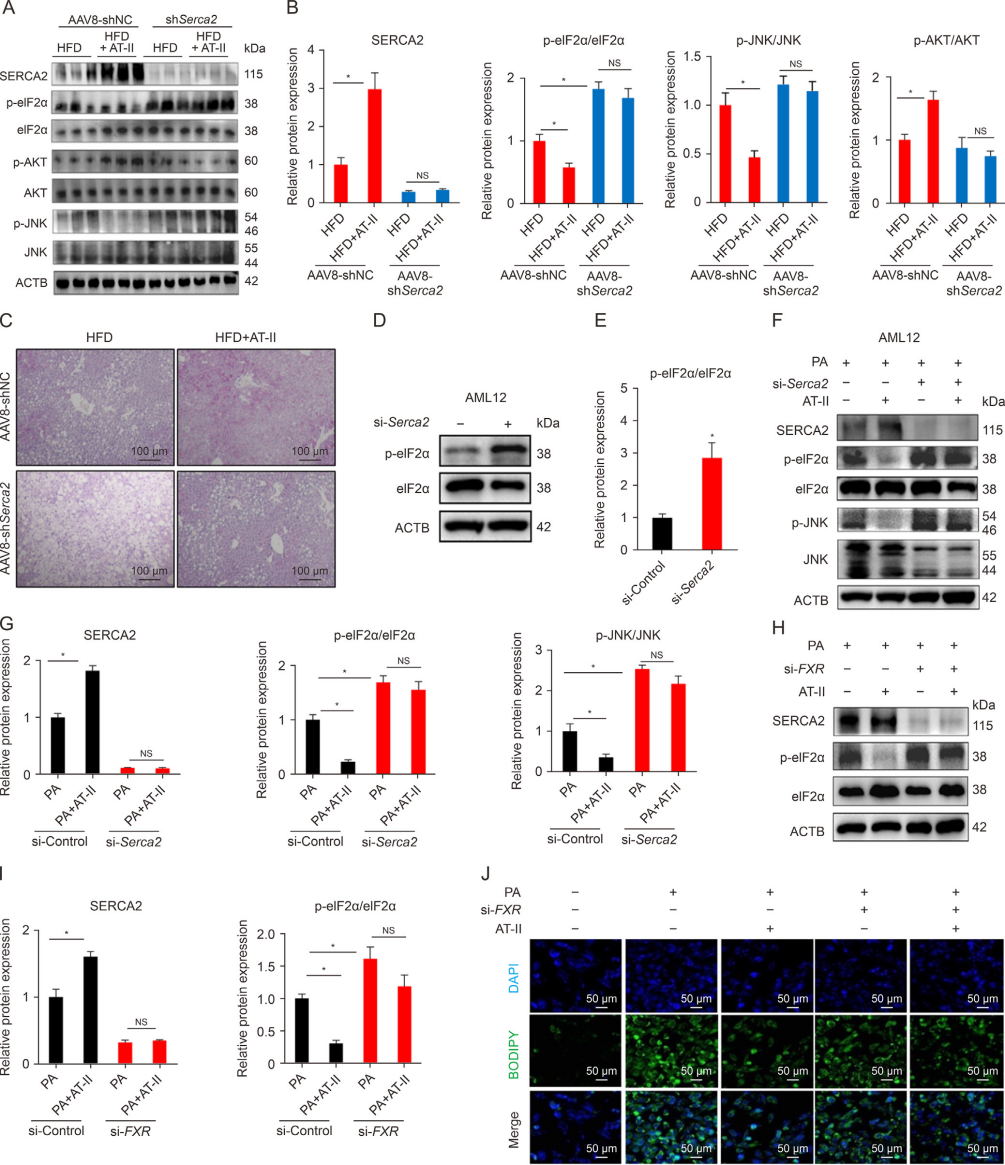
Sarco/endoplasmic reticulum Ca^2+^ ATPase 2 (SERCA2)-mediated eukaryotic translation initiation factor 2 subunit α (eIF2α) dephosphorylation in hepatocytes contributes to the anti-non-alcoholic fatty liver disease (NAFLD) effects of atractylenolide II (AT-II). (A–C) Obese male C57BL/6 mice injected with adeno-associated virus 8 (AAV8)-shNC and AAV8-sh*Serca2* via the tail vein injection were fed a high-fat diets (HFD) with or without AT-II (100 mg/100 g diet) for 6 weeks. *n* = 6. The liver tissues were collected for determination of protein expression by Western blotting (A), and quantified (B), and periodic acid-schiff (PAS) staining (C). (D, E) AML-12 cells were transfected with si-*Serca2* or si-Control for 72 h, and then subjected to intracellular eukaryotic translation initiation factor 2 subunit α (eIF2α) phosphorylation assessment by Western blotting (D), and quantified (E). *n* = 3. (F, G) AML-12 cells receiving si-*Serca2* or si-Control transfection were incubated with palmitic acid (PA) (400 μM) in the presence or absence of AT-II (50 μM) treatment for 24 h. The cells were collected for Western blotting analysis (F), and quantified (G). *n* = 3. (H, I) HepG2 cells with farnesoid X receptor (*FXR*)-RNAi were cultured with 400 μM of PA and PA + AT-II (50 μM) for 24 h *n* = 3. The cells were then subjected to Western blotting analysis (H), and quantified (I). (J) Representative images of lipid droplet boron-dipyrromethene (BODIPY) staining in HepG2 cells with or without *FXR* silencing after 24-h PA (400 μM) and PA + AT-II (50 μM) treatment. Image taken at ×400 magnification. All data were shown as the means ± standard error of the mean (SEM). ^∗^*P* < 0.05; NS: no significance. NC: negative control; AKT: protein kinase B; JNK: c-jun *N*-terminal kinase; ACTB: actin beta; DAPI: 4′,6-diamidino-2-phenylindole.

The aforementioned studies suggest that AT-II may alleviate ER stress by upregulating hepatic SERCA2, thereby mitigating fatty liver damage and improving hepatic insulin signaling. Hyperphosphorylation of hepatic eIF2α is a hallmark of ER stress and IR in the liver [29]. In next assays, we confirmed that AAV8-sh*Serca2* viral interference effectively reduced hepatic SERCA2 expression and prevented AT-II from inducing SERCA2 in the liver of mice (Figs. 10A and B). In mice injected with AAV8-shNC, AT-II significantly reduced the hepatic protein expression of p-eIF2α and p-JNK, while it increased p-AKT levels under HFD conditions (Figs. 10A and B), indicating an improvement in ER stress, liver damage, and insulin signaling. Notably, *Serca2* knockdown abolished these beneficial effects and even led to higher eIF2α phosphorylation compared to AAV8-shNC controls (Figs. 10A and B). Moreover, PAS staining demonstrated that AT-II significantly enhanced hepatic glycogen storage in obese mice, an effect that was not observed when hepatic SERCA2 was inhibited (Fig. 10C). These findings further supports the notion that hepatic dephosphorylation of eIF2α through restoration of SERCA2 is involved in AT-II's effects on improving hepatic ER stress and insulin signaling, contributing to improved hepatic glucose-lipid metabolism.

In line with the *in* *vivo* assays, we observed that silencing *Serca2* strongly induced eIF2α phosphorylation in normally AML-12 cells (Figs. 10D and E). Additionally, *Serca2* silencing also increased the protein levels of p-eIF2α and p-JNK, while it effectively blocked AT-II's ability to inhibit these proteins in AML-12 cells incubated with PA (Figs. 10F and G). Similarly, *FXR* knockdown also induced phosphorylation of eIF2α in clutured hepatocytes under PA insult (Figs. 10H and I). Meanwhile, *in vitro* silencing of *FXR* markedly impaired the effects of AT-II on SERCA2 upregulation and eIF2α dephosphorylation (Figs. 10H and I). These observations indicate that the modulation of SERCA2-eIF2α signaling by AT-II treatment requires the involvement of FXR. Furthermore, *FXR* silencing apparently attenuated the lipid-lowering effect of AT-II in an *in vitro* fatty liver cell model (Fig. 10J). Collectively, these findings suggest that the activation of FXR-SERCA2-eIF2α axis plays a crucial role in AT-II's capacity to restore normal hepatic ER stress signaling and insulin signaling, thereby combating NAFLD.

## 4 Discussion

In the present study, we demonstrated that SERCA2 is a novel downstream effector of FXR activation, which is essential for mediating hepatic eIF2α dephosphorylation and reducing ER stress. We also identified AT-II, a natural compound derived from AMK, as a novel FXR activator with potential effects on obesity and metabolic dysfunction, particularly fatty liver, in mice. Mechanistically, AT-II enhanced the hepatic FXR-SERCA2 axis, leading to eIF2α dephosphorylation and activation of insulin signaling. This, in turn, alleviated hepatic ER stress and IR, underscoring AT-II's therapeutic potential in NAFLD and related metabolic disorders.

Safety concerns such as dyslipidemia, pruritus, and liver toxicity have limited the use of certain prominent FXR agonist therapies, like OCA [30]. Moreover, different classes of FXR agonists have varied side effects, which may be linked to their different chemotypes [31]. Therefore, screening for novel FXR agonists among phytochemicals that differ structurally from those previously studied may help achieve therapeutic benefits while reducing side effects. Additionally, investigating the intricate molecular mechanisms underlying FXR agonist activity is crucial for fully understanding the positive effects of FXR activation. Previous studies have demonstrated that FXR activation negatively regulates ER stress by disrupting the PERK/eIF2α/CHOP pathway, thereby helping to control gastritis, liver injury, and fatty liver in mice [[20], [21], [22]]. These findings suggest that activating hepatic FXR may play a vital role in alleviating NAFLD. However, the precise mechanisms underlying this relationship require further investigation. Moreover, there has been limited attention given to the ability of phytochemicals to alleviate NAFLD by modulating FXR-ER stress signaling.

AT-II is a natural diterpene lactone isolated from Chinese medicinal herb AMK [23]. In this study, we identified AT-II as a novel FXR agonist through a series of *in vitro* molecular biology experiments including FXR transcriptional activity analysis and ligand-receptor binding assays. Furthermore, AT-II alleviated ER stress in hepatocytes induced by TM or PA, as evidenced by reduced levels of ER stress markers and the inhibition of the PERK/eIF2α/CHOP pathway activation. Additionally, AT-II improved lipid metabolism in HepG2 cells exposed to PA. The non-steroid structure of AT-II, along with its ability to activate FXR and alleviate ER stress in hepatocytes, promote us to investigate whether AT-II can effectively treat NAFLD by modulating the interplay between FXR and ER stress signaling pathways.

Defects in SERCA2 expression and activity contribute to ER stress and metabolic dysfunction [11,15]. Conversely, overexpression or pharmacological activation of SERCA2 can alleviate ER stress and reduce steatohepatitis in hepatocytes, while also improving systemic glucose homeostasis [13,14,32]. Interestingly, we found that both AT-II and GW4064 could enhance SERCA2 expression and activity in hepatocytes. In contrast, silencing FXR decreased SERCA2 expression in these cells, and *Serca2* expression was significantly lowered in *Fxr* knockout (*Fxr*^−/−^) mice compared to that in wild-type (WT) controls (Fig. S3). Subsequent experimental analyses verified that FXR activation directly regulated *SERCA2* expression at the transcriptional level. These results position SERCA2 as a critical mediator in the interaction between FXR and ER stress. However, a literature review reveals that few studies have investigated the role of FXR in the pathogenesis of liver disease through the modulation of SERCA2-mediated ER stress. This research gap underscores the necessity for further investigation into the potential of AT-II to mitigate ER stress and NAFLD *in vivo* by activating the FXR-SERCA2 pathway.

In our ongoing animal studies, we found that AT-II treatment effectively mitigated NAFLD in mouse models induced by HFD or MCD diets. This was achieved by improving IR and alleviating various liver conditions, including steatosis, fibrosis, inflammation, damage, apoptosis, oxidative stress, and ER stress. AT-II also activated the FXR-SERCA2 signaling and inhibited the PERK/eIF2α/CHOP pathway in the livers of DIO mice. Additionally, AT-II reduced fatty liver in mice treated with TM. In depth, silencing hepatic *Serca2* in DIO mice substantially diminished the positive effects of AT-II on metabolic disorders, particularly IR, hepatosteatosis, and liver injury. In fact, the inhibition of SERCA2 worsened liver ER stress, damage, and overall liver function. Furthermore, *FXR* knockdown in hepatocytes impaired the actions of AT-II in upregulating SERCA2 expression and reducing lipids over-accumulation. These results underscore the vital role of hepatic FXR-SERCA2 signaling in protecting the liver from ER stress-related injuries and in enabling AT-II to effectively combat NAFLD and IR. Importantly, AT-II treatment did not cause dyslipidemia in DIO mice; rather, it significantly lowered serum LDL-c levels while increasing HDL-c levels. This contrasts sharply with steroidal FXR agonists, such as OCA, which have opposing effects on these lipid markers in both clinical trials and animal studies [30]. Additionally, other nonsteroidal FXR agonists, such as GW4064 and LJN-452, have shown limited benefits in improving serum cholesterol levels [33,34].

NAFLD is closely linked with hepatic IR and JNK activation [35,36]. AKT, an essential component of insulin signaling, boosts insulin sensitivity and glycogen synthesis upon phosphorylation [37]. Previous studies suggest that enhancing hepatic glycogenesis can mitigate liver steatosis and IR [38,39]. Dephosphorylation of eIF2α, a critical regulator of ER stress and metabolic homeostasis, reduces liver glycogen levels, thereby improving glucose tolerance and hepatosteatosis in DIO mice [29]. Conversely, hyperphosphorylated eIF2α promotes apoptosis through JNK activation and AKT suppression [40]. Our results showed that silencing SERCA2 led to an increase in eIF2α phosphorylation in hepatocytes, thus resulting in JNK activation. Interestingly, AT-II induced eIF2α dephosphorylation in a SERCA2-dependent manner in both the liver tissues and hepatocytes. Additionally, AT-II enhanced hepatic AKT phosphorylation and glycogen storage while inhibited JNK activation. However, these effects were suppressed when SERCA2 was disrupted in the liver. These findings underscore the importance of SERCA2-mediated eIF2α dephosphorylation and hepatic insulin signaling in the efficacy of AT-II against NAFLD. Furthermore, *in vitro* knockdown studies showed that FXR was essential for AT-II's ability to promote SERCA2-mediated eIF2α dephosphorylation in hepatocytes. The results suggest that the activation of the FXR-SERCA2 pathway can promote eIF2α dephosphorylation in hepatocytes, allowing AT-II to improve NAFLD through modulating the interactions between FXR and ER stress.

Additionally, obesity is a pivotal factor in the development of NAFLD [41]. Studies have shown that OCA activates TGR5, which promotes the browning of adipose tissue and increases energy expenditure, leading to weight loss [42,43]. However, the activation of TGR5 is linked to pruritus, a significant side effect of OCA [44]. It is of concern that AT-II also counteracts obesity and promotes energy expenditure in DIO mice, accompanied by an increase in the expression of the thermogenic gene *Ucp1* in WAT. Despite this, our *in vitro* assays did not demonstrate that AT-II could activate TGR5 (Fig. S4). Both central and peripheral ER stress contribute to leptin resistance, which promotes energy imbalance, obesity, and NAFLD [45]. Recent research highlights the role of hepatic ER stress in cascading obesity and fatty liver disease, indicating that this stress can suppress adipose browning [46]. We also observed a significant decrease in serum leptin levels in AT–II–treated DIO mice, indicating leptin resistance was improved. Furthermore, SERCA2 activation has been linked to enhanced thermogenesis in fat tissue, aiding overall energy homeostasis. It is reasonable to hypothesize that AT-II's anti-obesity effects may result from its ability to alleviate ER stress. However, further investigation is needed to determine whether this beneficial action of AT-II on obesity is linked to hepatic FXR-SERCA2 signaling. The positive anti-obesity effects of AT-II is also confirmed by a recent study [25]. Thus, our findings suggest that AT-II may be a novel FXR agonist capable of improveing NAFLD and obesity by reducing ER stress.

Our study demonstrates that AT-II, a novel FXR agonist, can protect against NAFLD by addressing the interplay between FXR and ER stress, while AT-II therapy may help to avoid the negative effects of steroidal FXR agonists on lipid profiles. However, further investigation into the clinical efficacy and safety of AT-II in treating NAFLD is needed to enhance disease management and prevention strategies. Besides that, despite our *in vitro* assays showed that AT-II positively influenced anti-NAFLD effects and activated SERCA2 signaling through its FXR agonism, we could not exclude potential off-target effects *in vivo* due to the absence of experiments using FXR knockout mice. Moreover, the unique effects of AT-II on SERCA2 transcription and its subsequent metabolic pathways in comparison to other FXR agonists, such as GW4064, warrant deeper investigation. The differences in transcriptional pattern among FXR agonists with varying structures could be significant. Additionally, future research should explore potential limitations of using AT-II as an FXR activation strategy, particularly regarding its impact on bile acid dysregulation.

## 5 Conclusions

In summary, the present study revealed AT-II mitigates ER stress-related fatty liver disease through targeting the FXR-SERCA2-eIF2α signaling pathway, and emphasized the importance of the interplay between FXR and ER stress in controlling NAFLD. Our results suggest that AT-II could be a promising therapeutic option for managing NAFLD and its associated metabolic disorders.

## CRediT authorship contribution statement

**Ming Gu:** Writing – original draft, Methodology, Investigation, Funding acquisition, Data curation, Conceptualization, Formal analysis. **Zhiwei Chen:** Investigation, Methodology, Data curation, Validation. **Yujun Chen:** Investigation, Formal analysis, Validation. **Yiping Li:** Investigation. **Hongqing Wang:** Investigation. **Ya-ru Feng:** Investigation. **Peiyong Zheng:** Conceptualization, Resources, Supervision. **Cheng Huang:** Supervision, Resources, Project administration, Conceptualization, Writing – review & editing.

## Data availability statement

Data information can be provided upon a reasonable inquiry.

## Declaration of competing interest

The authors declare that there are no conflicts of interest.
