# Supplementary material for: Unlocking the potential of atractylenolide II: Mitigating non-alcoholic fatty liver disease through farnesoid X receptor-endoplasmic reticulum stress interplay
Source: J Pharm Anal. 2025 Apr 21;15(12):101318. doi: 10.1016/j.jpha.2025.101318 (PMC12765252; doi:10.1016/j.jpha.2025.101318)
Supplement: Multimedia component 1 [file mmc1.docx]

Supplementary file

Unlocking the potential of atractylenolide II: mitigating non-alcoholic fatty liver disease through farnesoid X receptor-endoplasmic reticulum stress interplay

**Supplementary figures**


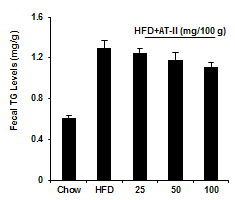


**Figure S1.** Atractylenolide II (AT-II) has no effect on mouse fecal total triglycerides (TG) excretion. Obese male C57BL/6 mice were fed a high-fat diets (HFD) with or without AT-II (25, 50, or 100 mg/100 g diet) for 6 weeks. Fecal total TG content was determined using commercial kits (Nanjing Jiancheng, Nanjing, China) according to the manufacturers' protocols. *n* = 7. The data showed that AT-II had no influence on the fecal total TG excretion. All data are statistically analyzed as the means ± standard error of the mean (SEM).


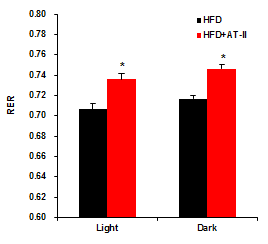


**Figure S2.** Atractylenolide II (AT-II) treatment increases mouse respiratory exchange ratio (RER). Diet-induced obesity (DIO) C57BL/6 mice were fed a high-fat diets (HFD) with or without AT-II (100 mg/100 g diet) for 6 weeks. Then, the mice were housed in metabolic cages to measure metabolic parameters, mouse respiratory exchange ratio (RER) during 24 h was caculated by using the Columbus Instruments Comprehensive Lab Animal Monitoring System (CLAMS, Columbus Instruments, Ohio, USA). *n* = 5. **P* < 0.05, HFD vs HFD+AT-II group. All data are statistically analyzed as the means ± standard error of the mean (SEM).


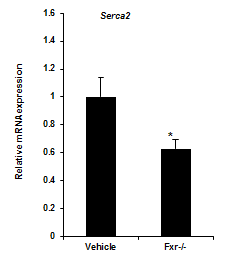


**Figure S3.** Farnesoid X receptor (*Fxr*) knockout reduces hepatic sarco/endoplasmic reticulum Ca^2+^ ATPase 2 (*Serca2*) expression. livers from 15-week-old wild type male C57BL/6 mice as wild type (WT) vehicle and Fxr^-/-^ mice fed chow diet were used to analyze relative mRNA expression of *Serca2*. Data are statistically analysed as the means ± standard error of the mean (SEM) (*n* = 6). **p* < 0.05, *vs*. vehicle group. Actin beta (*Actb*) was used as an internal control for normalizing the messenger RNA (mRNA) levels.


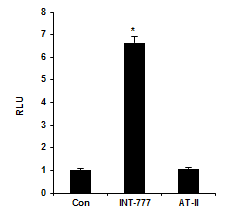


**Figure S4.** Atractylenolide II (AT-II) had no impact on G protein-coupled bile acid receptor 1 (TGR5) activity. HEK293T cells were plated in 48-well plates and cultured for 24 h prior to transfection. Then, 2 μg of each expression plasmids for phTGR5, CRE-Luc, and 0.2 μg of internal interference plasmid pREP7 Renilla luciferase were co-transfected into HEK293T cells by using the lipofectamine 3000 kit. Then cells were treated with 0.1% dimethyl sulfoxide (DMSO), INT-777 (10 μM) and AT-II (50 μM) for an additional 24 hours before detection of TGR5 activity. Data are statistically analysed as the means ± standard error of the mean (SEM) (*n* = 3). **P* < 0.05, *vs*. Control group. Renilla luciferase activity served as a normalization control for transfection efficiencies. All data of relative light unit (RLU) were normalised to the mean of the control group. All transfection assays were carried out in triplicate with three independent repeats.

**Table S1． Sequences of the human primers used in quantitative real-time polymerase chain reaction**

| Gene Sense primer Anti-sense primer |
| --- |
| *FXR* ATGGGAATGTTGGCTGAATG CCTGCATGACTTTGTTGTCG  *SHP* AGGCCTCCAAGCCGCCTCCCACATTGGGC GCAGGCTGGTCGGAAACTTGAGGGT  *ABCC2* TCGGAATGTGAATAGCCTGAAG CGCAAGGATGATGAAGAATATCG  *ACOX1* CCAAGCTTTCCTGCTCAGTGTT CCCCCAGTCCCTTTTCTTCA  *PPARα* GAAATGGGAAACATCCAAGAGA CACAGGATAAGTCACCGAGGA  *CYP7A1* GAGAAGGCAAACGGGTGAAC AGCACAGCCCAGGTATGGA  *CPT1α* TGGCGTCTGAGAAGCATCAGCATA ACACCACGTAAAGGCAGAAGAGGT  *APOC2* GAGATGCCTAGCCCGACCTTCCTCAC GCTCAGTCTGAACCTGGGGGATCAGG  *SERCA2* TGGGTGTATGGCAGGAAAGAA ACTGGTCAACTCTTAGTGTGGTA  *BIP* TCCTATGTCGCCTTCACT ACAGACGGGTCATTCCAC  *CHOP* GGAAACAGAGTGGTCATTCCC CTGCTTGAGCCGTTCATTCTC  *ATF4* ATGACCGAAATGAGCTTCCTG GCTGGAGAACCCATGAGGT  *ERDJ4* TCTTAGGTGTGCCAAAATCGG TGTCAGGGTGGTACTTCATGG  *ACTB* AATCTGGCACCACACCTTCTA ATAGCACAGCCTGGATAGCAAC |

FXR: farnesoid X receptor; SHP: nuclear receptor subfamily 0, group B, member 2; ABCC2: ATP-binding cassette sub-family C member 2; ACOX1: acyl-CoA oxidase 1; CPT1α: carnitine palmitoyltransferase 1 α; BIP: glucose-regulated protein 78; ERDJ4: endoplasmic reticulum dnaJ heat shock protein 4; ATF4: activating transcription factor 4; CHOP: C/EBP Homologous Protein; CYP7A1: cytochrome p450 family 7 subfamily a member 1; PPARα: proliferator-activated receptor α; APOC2: apolipoprotein C-II; SERCA2: sarco/endoplasmic reticulum Ca^2+^ ATPase 2; ACTB: actin beta.

**Table S2． Sequences of the mouse primers used in quantitative real-time polymerase chain reaction**

| Gene | Sense primer | Anti-sense primer |
| --- | --- | --- |
| *Actb* | TGTCCACCTTCCAGCAGATGT | AGCTCAGTAACAGTCCGCCTAGA |
| *Fxr* | TTCCTCAAGTTCAGCCACAG | TCGCCTGAGTTCATAGATGC |
| *Shp* | GGAGTCTTTCTGGAGCCTTG | ATCTGGGTTGAAGAGGATCG |
| *Cpt1α* | TATGTGAGTGACTGGTGGGAGGA | TATGGGTTGGGGTGATGTAGAGC |
| *Pparα* | AGGCTGTAAGGGCTTCTTTCG | GGCATTTGTTCCGGTTCTTC |
| *Cebpα* | CGCAAGAGCCGAGATAAAGC | CACGGCTCAGCTGTTCCA |
| *Cebpβ* | CTGCGGGGTTGTTGATGT | ATGCTCGAAACGGAAAAGGT |
| *Gpx* | CCTTTTAAGCAGTATGCAGGCA | CAAGCCAAATGGCCCAAGTT |
| *Gclc* | GGGGTGACGAGGTGGAGTA | GTTGGGGTTTGTCCTCTCCC |
| *Sod2* | CAGACCTGCCTTACGACTATGG | CTCGGTGGCGTTGAGATTGTT |
| *Serca2* | TGTTTGTCCTATTTCGGGGTG | AATCCGCACAAGCAGGTCTTC |
| *Scd1* | CTTATCATTGCCAACACCA | CTTCTCGGCTTTCAGGTC |
| *Tnfα* | ATGGATCTCAAAGACAACCAACTAG | ACGGCAGAGAGGAGGTTGACTT |
| *Mcp-1* | AGGTCCCTGTCATGCTTC | GTGCTTGAGGTGGTTGTG |
| *Il1β* | TCGTGCTGTCGGACCCATAT | GGTTCTCCTTGTACAAAGCTCATG |
| *Il6* | AACCACGGGCTTCCCTACTT | TCTGTTGGGAGTGGTATCCTCTGT |
| *Cyp7a1* | TGATCCTCTGGGCATCTCAAGCAA | AGCTCTTGGCCAGCACTCTGTAAT |
| *Abcb4*  *Bip* | CGGCGACTTTGAACTAGGCA  ACTTGGGGACCACCTATTCCT | CAGAGTATCGGAACAGTGTCAAC  GTTGCCCTGATCGTTGGCTA |
| *Chop*  *Cpt1β* | CTCGCTCTCCAGATTCCAGTC  TGGGACTGGTCGATTGCATC | CTTCATGCGTTGCTTCCCA  CAGGGTTTGTCGGAAGAAGAAAA |
| *Ucp1* | CATCACCACCCTGGCAAAA | AGCTGATTTGCCTCTGAATGC |
| *Ucp2* | GGGCACTGCAAGCATGTGTA | TCAGATTCCTGGGCAAGTCACT |
| *Ucp3*  *Abcc2* | TGGCCCAACATCACAAGAAA  GTGTGGATTCCCTTGGGCTTT | TCCAGCAACTTCTCCTTGATGA  CACAACGAACACCTGCTTGG |
| *Col1α1*  *Tgfβ*  *α-Sma*  *Srebp1c*  *Fas*  *Acox1*  *Cd36*  *Abcb11* | CATGAGCCGAAGCTAACCC  CCACCTGCAAGACCATCGAC  GCAGGGAGTAATGGTTGGAAT  GGCTATTCCGTGAACATCTCCTA  CTGAGATCCCAGCACTTCTTGA  CTTGGATGGTAGTCCGGAGA  GGAGCCATCTTTGAGCCTTCA  CGGACCTGTATTGTCATTGC | TGTGGCAGATACAGATCAAGC  CTGGCGAGCCTTAGTTTGGAC  TCTCAAACATAATCTGGGTCA  ATCCAAGGGCATCTGAGAACTC  GCCTCCGAAGCCAAATGAG  TGGCTTCGAGTGAGGAAGTT  GAACCAAACTGAGGAATGGATCT  CCCTTCTGGTCCATCAGTTT |

**Table S3．Predicted farnesoid X receptor binding sites on the human sarco/endoplasmic reticulum Ca^2+^ ATPase 2 promoter**

| Target site | Sequence |
| --- | --- |
| Site#1: -1360~-1350  Site#2: -1789~-1779  Site#3: -1993~-1983 | GAAATGGCCTT  TAAATGACATG  TCCATGACCAC |

**Table S4． Primers for the amplification of the promoter region of human sarco/endoplasmic reticulum Ca^2+^ ATPase 2 using real-time PCR**

| Forward primer | Reverse primer |
| --- | --- |
| TCCACCTGGCTTCTCTCAGT | TCAGGCACGTCATGTCATTT |

**Table S5．siRNA sequence**

| Target human *FXR* | Target mouse *Serca2* |
| --- | --- |
| si-*FXR*:  5′-GAGGAUGCCUCAGGAAAUA-3′  si-Control:  5′-AAAGCGUCUGGAAAAGUCG-3′ | si-*Serca2*:  5′-UGACUCUGCUUUGGAUUAUAA-3′  si-Control:  5′-UUCUCCGAACGUGUCACGU-3 |

**Table S6．Adeno-associated virus 8 (AAV8)-shRNA sequence for mouse hepatic sarco/endoplasmic reticulum Ca^2+^ ATPase 2 knock down**

| shRNA sequence |
| --- |
| AAV8-shNC: 5’-GATCTGTTCTCCGAACGTGTCACGTTTCAAGAGAACGTGACACGTTCGGAGAATTTTTTC-3’  AAV8-sh*Serca2*: 5’-GATCCGTGACTCTGCTTTGGATTATAACTCGAGTTATAATCCAAAGCAGAGTCATTTTTT-3’ |
